# Cardiovascular Therapy Benefits of Novel Antidiabetic Drugs in Patients With Type 2 Diabetes Mellitus Complicated With Cardiovascular Disease: A Network Meta‐Analysis

**DOI:** 10.1111/1753-0407.70044

**Published:** 2025-01-09

**Authors:** Saixian Shi, Xiaofeng Li, Ye Chen, Jiahao Li, Yan Dai

**Affiliations:** ^1^ School of Pharmacy Southwest Medical University Luzhou Sichuan Province China; ^2^ Pangang Xichang Hospital Xichang Sichuan Province China; ^3^ Department of Pharmacy Affiliated Hospital of Southwest Medical University Luzhou Sichuan Province China

**Keywords:** cardiovascular outcomes, dipeptidyl peptidase 4 inhibitor, glucagon‐like peptide 1 receptor agonist, sodium‐glucose cotransporter 2 inhibitor, Type 2 diabetes mellitus

## Abstract

**Objective:**

Provide an evidence‐based basis for the selection of cardiovascular benefit drugs in Type 2 diabetes mellitus (T2DM) patients with cardiovascular disease (CVD).

**Methods:**

Conduct a comprehensive search of all relevant literature from PubMed, Embase, Web of Science, Cochrane Library, and Clinical Trials.gov from their establishment until December 13, 2023, and select randomized controlled trials (RCTs) that meet the pre‐established inclusion and exclusion criteria. Use the Cochrane bias risk assessment tool to evaluate the quality of the included literature. Use R 4.3.2 software to conduct network meta‐analysis for drug category comparison.

**Results:**

A total of 24 large‐scale randomized controlled trials (RCTs) were included, including 19 intervention measures, and 172 803 patients participated in the study. The results of the network meta‐analysis show that: GLP1RA (OR 0.89, 95% CI 0.81–0.97) and SGLT2i (OR 0.91, 95% CI 0.83–0.99) can reduce the occurrence of major adverse cardiovascular events (MACE), GLP1RA (OR 0.88, 95% CI 0.79–0.97) and SGLT2i (OR 0.89, 95% CI 0.81–0.99) reduced the risk of cardiovascular death. SGLT2i (OR 0.68, 95% CI 0.62–0.75) reduced the occurrence of hospitalization for heart failure, GLP1RA (OR 0.88, 95% CI 0.81–0.97) and SGLT2i (OR 0.89, 95% CI 0.80–0.97) reduced the occurrence of all‐cause death.

**Conclusion:**

In the comparison of new hypoglycemic drug classes, GLP1RA and SGLT2i reduced MACE, cardiovascular mortality and all‐cause mortality in T2DM patients with CVD, with no significant difference in efficacy, and DPP4i was noninferior to placebo. Only GLP1RA reduced the risk of nonfatal stroke, and only SGLT2i reduced the risk of HHF.

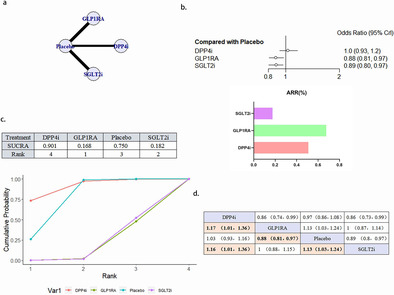

**Abbreviations**
ARR: Absolute Risk Reduction
BMI: Body mass index
CI: Confidence Intervals
CVD: Cardiovascular disease
DPP‐4: Dipeptidyl peptidase 4
DPP4i: Dipeptidyl peptidase 4 inhibitors
eGFR: Glomerular filtration rate
FDA: Food and Drug Administration
GBD: Global Burden of Diseases, Injuries, and Risk Factors Study
GLP‐1: Glucagon‐like peptide 1
GLP1RA: Glucagon‐like peptide 1 receptor agonist
HF: Heart failure
HFrEF: Heart failure with reduced ejection fraction
HHF: Hospitalization for heart failure
MACE: Major adverse cardiovascular events
MCMC: Markov chain Monte Carlo
OR: Odds Ration
PSRF: Potential scale reduction factor
RCTs: Randomized controlled trials
SDF‐1α: stromal cell‐derived factor‐1alpha
SGLT‐2: Sodium‐glucose cotransporter protein 2
SGLT2i: Sodium‐glucose cotransporter protein 2 inhibitors
SUCRA: Surface under the cumulative ranking curve
T2DM: Type 2 Diabetes Mellitus

**Summary**

- The prevalence of diabetes is increasing year‐by‐year, and its cardiovascular complications seriously endanger the health of citizens, leading to a huge medical burden.
- The purpose of this study was to evaluate the effects of dipeptidyl peptidase 4 inhibitor (DPP4i), glucagon like peptide 1 receptor agonist (GLP1RA), and sodium‐glucose cotransporter protein 2 inhibitors (SGLT2i), on cardiovascular outcome events in patients with Type 2 diabetes mellitus (T2DM) cardiovascular disease (CVD), provide an evidence‐based basis for the selection of cardiovascular benefit drugs in T2DM patients with CVD.

## 1 Introduction

Diabetes mellitus is a chronic metabolic disease characterized by persistent hyperglycemia. The most basic cause of its disease is insufficient insulin secretion or utilization disorder, which greatly endangers public health worldwide and causes great medical expenses worldwide [1]. Its prevalence is increasing year‐by‐year. According to GBD (Global Burden of Diseases, Injuries and Risk Factors Study) results, the prevalence of age‐standardized diabetes worldwide in 2021 was 6.1%, and the number of patients reached 529 million [2]. GBD used the social‐demographic index and body mass index (BMI) as predictors of the number of diabetes worldwide in 2050, a figure that could reach an staggering 1.31 billion [2]. Diabetes mellitus and its complications are one of the main causes of death and disability worldwide, and further breakthroughs are needed in the diagnosis and treatment and management of diabetes mellitus [2].

According to its different causes, diabetes is mainly divided into Type 1 diabetes, Type 2 diabetes mellitus (T2DM), gestational diabetes, monogenic diabetes, secondary diabetes, and stress diabetes [3]. Among these, T2DM was the most common. According to the GBD data, T2DM cases accounted for about over 90% of all types of diabetes cases worldwide in 2021 [2]. The most terrible thing about diabetes is a series of complications due to long‐term hyperglycemia. Long‐term hyperglycemia symptoms can lead to microvascular and large vascular lesions, involving the heart, brain, kidney, peripheral nerves, eyes, feet and other systems [4]. Cardiovascular disease (CVD) is a very common and serious complication in diabetic patients, and it is also one of the main culprits of death in diabetic patients [5, 6]. The American Society of Cardiology first proposed the idea that “diabetes is a cardiovascular disease” in 1999 [7]. Compared with nondiabetic patients, diabetic patients have an increased risk of CVD, mainly including coronary artery disease, heart failure (Heart failure, HF), atrial fibrillation, cerebrovascular disease, aorta and peripheral artery disease [8, 9]. Obesity, dyslipidemia, elevated blood pressure, smoking and alcohol consumption may aggravate the risk of CVD in diabetic patients [6]. If the main risk factors for CVD in T2DM patients can be effectively controlled within the target range in the long term, the risk of CVD and death is very small [6]. Unfortunately, only 5%–6% of people with diabetes manage CVD risk factors [10].

The treatment of diabetes includes the use of hypoglycemic drugs as well as some nonpharmacological therapies, including diet therapy, exercise therapy, psychotherapy, acupuncture therapy, and more [11]. Classical hypoglycemic drugs include insulin, metformin, sulfonylurea insulin promoter, α‐glycosidase inhibitors, glinide insulin promoter, thiazolidinedione insulin sensitizer [12] With the continuous updating of the concept of diabetes treatment, new hypoglycemic drugs have been developed and successively applied in diabetic patients. The new hypoglycemic agents include DPP4i (Dipeptidyl peptidase 4 inhibitors), GLP1RA (Glucagon‐like peptide‐1 receptor agonist), and SGLT2i (Sodium‐glucose cotransporter protein 2 inhibitors).

The glucose‐lowering effects of both DPP4i and GLP1RA were based on the GLP‐1 (glucagon‐like peptide 1) [13, 14]. DPP4i maintains the hypoglycemic effect of GLP‐1 secreted in vivo by inhibiting the degradation of GLP‐1 by DPP‐4 (Dipeptidyl peptidase 4) [15]. The representative drugs sitagliptin, linagliptin, vilagliptin, saxagliptin, and alogliptin are widely used around the world, and their hypoglycemic effect is widely recognized, with relatively good drug tolerance and safety [16]. DPP4i appears to have some positive cardiovascular effects in preliminary studies of DPP4i [17]. However, in subsequent cardiovascular outcome experiments, sitagliptin [18], Alogliptin [19] Linagliptin [20] showed noninferiority in patients with T2DM and high cardiovascular risk, but were not statistically superior to placebo, while Saxagliptin was found to potentially increase the risk of HF [21]. The cardiovascular protective role of DPP4i is still controversial.

GLP1RA activates the GLP‐1 receptors in the human body, effectively improving overall blood glucose levels by increasing glucose utilization in muscle and adipose tissue and reducing hepatic glucose release. Representative drugs are Liraglutide, Semaglutide, Dulaglutide, Albiglutide, exenatide, Lixisenatide, and others [22]. Results of several clinical trials showed the cardiovascular protective effects of GLP1RA in patients with T2DM [23, 24, 25, 26]. However, there are no clinical trials based on direct comparisons between GLP1RA drugs.

SGLT2i reduces blood glucose by inhibiting SGLT‐2 in the proximal convoluted sub‐tube in the kidney and inhibiting the renal reabsorption of glucose [27]. Representative drugs are Dapagliflozin, Empagliflozin, and Canagliflozin. Compared with other hypoglycemic drugs, SGLT2i has multiple effects such as hypoglycemic, weight loss and antihypertensive, has fewer adverse reactions, has good oral bioavailability and hypoglycemic persistence, and shows cardiovascular protective effects in many large cardiovascular outcome trials [28, 29, 30].

The high risk of diabetes combined with CVD has a great impact on the survival rate, quality of life, and prognosis of patients. Therefore, the choice of anti‐diabetic treatment strategy should not be limited to the improvement of HbA 1 c levels, but should pay more attention to the cardiorenal risk and long‐term cardiovascular benefits of individual patients. Most of the existing clinical trials are placebo‐controlled trials, which lack a direct comparison between specific novel hypoglycemic drugs. Network meta‐analysis can combine direct comparison and indirect comparison to achieve the comparison of efficacy and ranking of multiple interventions. This study conducted a systematic review and network meta‐analysis based on existing clinical randomized controlled experiments to evaluate the efficacy differences of DPP4i, GLP1RA, and SGLT2i in T2DM patients with CVD, in order to provide evidence‐based basis for the selection of clinical treatment options for T2DM patients with CVD.

## 2 Methods

All studies included in this meta‐analysis followed the principles of the Helsinki Declaration of the World Medical Association, and studies involving human subjects obtained informed consent from the subjects and were reviewed and approved by the Ethics Committee of the Affiliated Hospital of Southwest Medical University (license number: 2024‐0013).

### 2.1 Literature Search

Five databases of Pubmed, Cochrane Library, Embase, Web of Science, and Clinical Trials.gov were used to search comprehensively, from the database establishment to all relevant literature published on December 13, 2023. The search term is “Dipeptidyl peptidase 4 inhibitor,” “sitagliptin,” “sitagliptin phosphate,” “sitagliptin,” “linagliptin,” “vildagliptin,” “saxagliptin,” “alogliptin,” “gemigliptin,” “dutogliptin,” “anagliptin,” “evogliptin,” “gosogliptin,” “omarigliptin,” “teneligliptin,” “trelagliptin,” “glucagon like peptide 1 receptor agonist,” “exenatide,” “liraglutide,” “lixisenatide,” “semaglutide,” “dulaglutide,” “albiglutide,” “sodium glucose cotransporter 2 inhibitor,” “canagliflozin,” “dapagliflozin,” “empagliflozin,” “ertugliflozin,” “ipragliflozin,” “sotagliflozin,” “tofogliflozin,” “luseogliflozin,” “remogliflozin,” “sergliflozin,” “diabetes mellitus, Type 2,” “Cardiovascular Diseases,” “Acute Coronary Syndrome,” “Heart Failure,” “Coronary Disease,” “Atherosclerosis,” “Arrhythmias, Cardiac,” “Atrial Fibrillation,” “Ventricular Fibrillation,” “Angina Pectoris,” “Myocardial Infarction,” “Hypertension,” “Cardiomyopathies,” “Diabetic Cardiomyopathies,” “Stroke,” “randomized,” and its synonym. The complete search formula of each database is shown in Table S1.

### 2.2 Inclusion and Exclusion Criteria

The inclusion criteria were developed following the PICOS principle, and the included studies met the following criteria:

1. Population: adult patients with T2DM and cardiovascular system diseases, including coronary artery disease, myocardial infarction, cerebrovascular disease, peripheral vascular disease, HF, hypertension, arrhythmia.
2. Interventions: Give any one of the new hypoglycemic drugs (including DPP4i, GLP1RA, SGLT2i) based on the conventional hypoglycemic treatment regimen.
3. Comparison: placebo or any new hypoglycemic drug based on conventional therapy.
4. Outcome: MACE (Major adverse cardiovascular events, including cardiovascular death, nonfatal myocardial infarction or nonfatal stroke events), cardiovascular death, nonfatal myocardial infarction, nonfatal stroke, HHF (Hospitalization for heart failure) and all‐cause mortality.
5. Study design: RCT (Randomized controlled trials). Articles in English or translated to English.

Exclusion criteria:

1. Uncompleted or completed clinical studies with no valid outcome data, outcome indicators do not meet or cannot provide complete data.
2. Non‐RCTs, including retrospective, case–control, case reports, cohort, cross‐sectional studies.
3. Repeated publication of the literature.

### 2.3 Article Sieving and Data Extraction

Two researchers will independently complete the literature screening and data extraction, and finally cross‐check. If there is any disagreement, both parties will discuss or request a third party to decide.

First, duplicate documents were removed, and the first screening was completed by browsing the literature titles and abstracts, excluding obviously inconsistent documents. Second, all the full text and its materials after the initial screening were obtained and read carefully, and the literature after the initial screening were re‐screened again to determine the final included literature according to the inclusion and exclusion criteria. The Cochrane Quality Evaluation table was used to evaluate the risk of bias of the included literature and to map the risk of bias using R 4.3.2 software.

Extracted information includes: (1) general information: name of the first author, year of publication of the literature, study design, study sample size, trial name, and clinical trial registration number. (2) Baseline characteristics of the study subjects, including mean age, sex distribution, physical mass index (BMI) level, duration of diabetes, HbA 1 c, level, and combined CVD. (3) Study protocol: Specific interventions, including drug name, dose, administration method, intervention time, and follow‐up time. (4) Outcome measures: MACE, cardiovascular death, nonfatal myocardial infarction, nonfatal stroke, HHF, number of all‐cause deaths and the total sample size used to analyze this outcome measure.

### 2.4 Statistical Analysis

Network meta‐analysis was performed using the R 4.3.2 software. The outcome index were dichotomous variables, OR (odds ratio) was used as the effect size, and the 95% CI (confidence interval) was calculated. A Bayesian random effects model using the MCMC (Markov chain Monte Carlo) was used to achieve indirect comparisons between interventions. The comparative relationship between all interventions was visually displayed by drawing the network graph. Mapping the forest plot of each intervention versus placebo. In addition, ARR (absolute risk reduction) was calculated. SUCRA (Surface under the cumulative ranking curve), bar chart of sorted probability and the Line plots of the cumulative probabilities represent the effectiveness ranking of various treatment methods. A league table presents the results of the comparison between each two interventions, using effect values OR and 95% CI to determine whether the pairwise comparisons between interventions were statistically different. Publication bias was assessed using the STATA 14 draw comparison‐adjusted funnel plot.

## 3 Results

### 3.1 Retrieval Results of Literature

**FIGURE 1:**
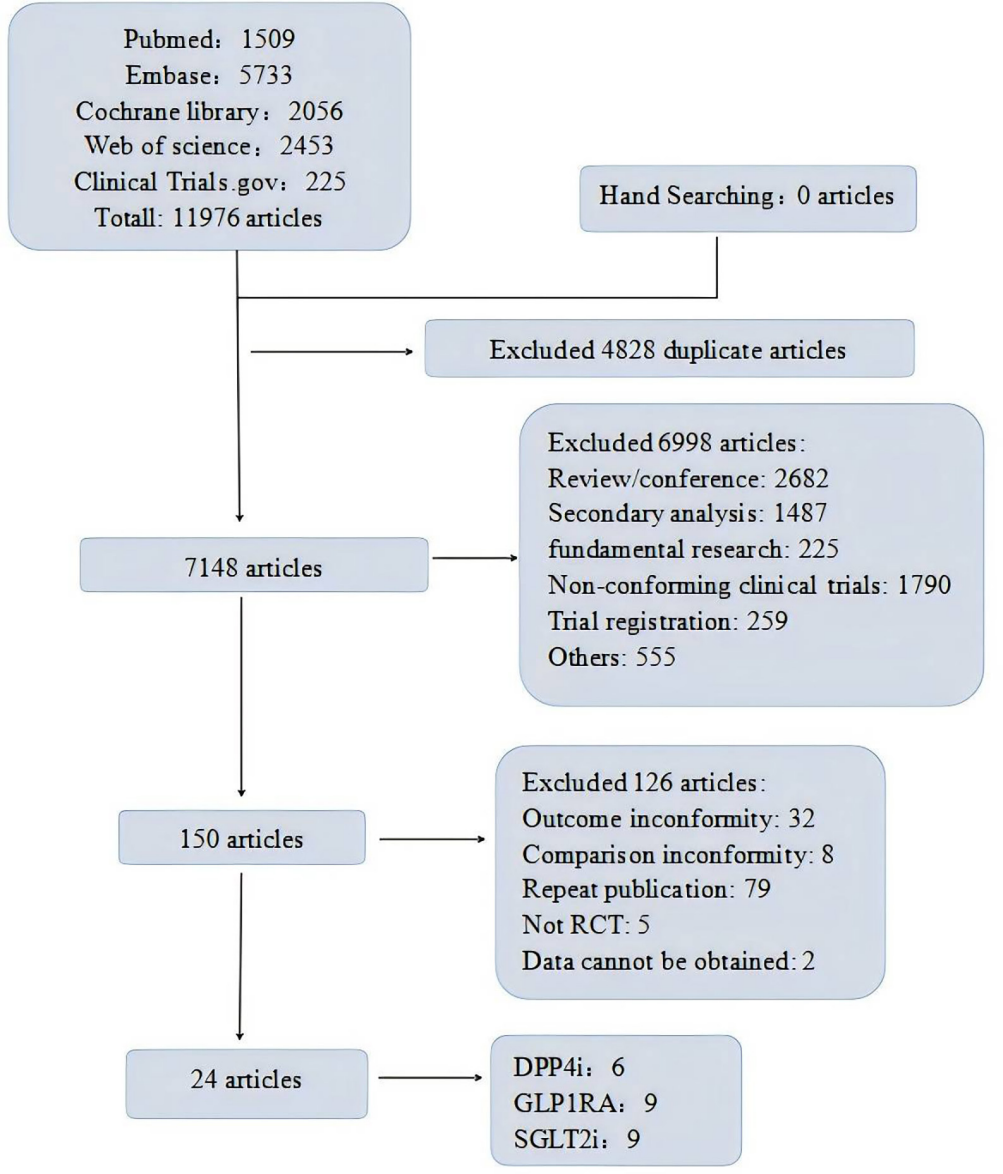
Flow chart of the literature search and screening.

A total of 11 976 articles were retrieved from Pubmed (*n* = 1509), Cochrane Library (*n* = 2056), Embase, Web of science (*n* = 2453), and Clinical Trials.gov (*n* = 225). Four thousand eight hundred and twenty‐eight duplicate references were removed. Read the names and abstracts of the initial literature and exclude the clearly inconsistent literature. Read the full text, exclude the documents with inconsistent outcome indicators, inconsistent control measures, repeated publication, incomplete data or inaccessible data, and finally select 24 literatures that met the inclusion and exclusion criteria, including 6 articles related to DPP4i, 9 articles related to GLP1RA, and 9 articles related to SGLT2i. Literature screening flow diagram is shown in Figure 1.

### 3.2 Baseline Characteristics

Of the 24 included articles, involved 19 interventions and a total of 172 803 patients. One articles reported results of Saxagliptin (SAVOR‐TIMI 53, NCT01107886 [21]), One articles reported results of Sitagliptin (TECOS, NCT00790205 [18]), One articles reported results of Alogliptin (EXAMINE, NCT00968708 [19]), One articles reported results of Vildagliptin (VIVIDD, NCT00894868 [31]), One articles reported results of Omarigliptin (NCT01703208 [32]), One articles reported results of Linagliptin (CARMELINA, NCT01897532 [20]), One articles reported results of Albiglutide (Harmony Outcomes, NCT02465515 [23]), One articles reported results of Liraglutide (LEADER, NCT01179048 [24]), One articles reported results of Efpeglenatide (AMPLITUDE‐O, NCT03496298 [33]), One articles reported results of Dulaglutide (REWIND, NCT01394952 [25]), One articles reported results of Semaglutide (PIONEER 6, NCT02692716 [26]; SUSTAIN‐6, NCT01720446 [34]), One articles reported results of Lixisenatide (ELIXA, NCT01147250 [35]), Two articles reported results of exenatide (NCT01455896 [36], EXSCEL, NCT01144338 [37]), One articles reported results of Ertugliflozin (VERTIS CV, NCT01986881 [38]), Two articles reported results of Canagliflozin (CANVAS, NCT01032629和NCT01989754 [30]; CREDENCE, NCT02065791 [39]), Two articles reported results of Dapagliflozin (DECLARE–TIMI 58, NCT01730534 [28]; DAPA‐HF, NCT03036124 [40]), Two articles reported results of Empagliflozin (EMPA‐REG OUTCOME [41], NCT01131676; EMPEROR‐Reduced, NCT03057977 [42]), Two articles reported results of Sotagliflozin (SOLOIST‐WHF, NCT03521934 [43]; SCORED, NCT03315143 [44]). All control measures included in the literature were placebo with a median follow‐up up to 5.4 years. All patients had T2DM complicated with CVD, including coronary artery disease, hypertension, myocardial infarction, HF, peripheral vascular disease, and stroke. Basic characteristics of the included literature are described in Tables 1 and 2.

**TABLE 1:** Basic characteristics of the included literature.

Study	Intervention	Administration	Sample size	Age (year)	Female (*n*, %)	BMI (kg/m^2^)	Median follow‐up time
Scirica etal., 2013 [21]	Saxagliptin	2.5–5 mg, qd, po	8280	65.1 ± 8.5	2768 (33.4)	31.1 ± 5.5	2.1 years
	Placebo		8212	65.0 ± 8.6	2687 (32.7)	31.2 ± 5.7	2.1 years
Green et al., 2015 [18]	Sitagliptin	50–100 mg, qd, po	7332	65.4 ± 7.9	2134 (29.1)	30.2 ± 5.6	3 years
	Placebo		7339	65.5 ± 8.0	2163 (29.5)	30.2 ± 5.7	3 years
White et al., 2013 [19]	Alogliptin	6.25–25 mg, qd, po	2701	61.0	873 (32.3)	28.7 ± 10.05	1.5 years
	Placebo		2679	61.0	856 (32.0)	28.7 ± 13.175	1.5 years
McMurray et al., 2018 [31]	Vildagliptin	50 mg, qd or bid, po	128	62.9 ± 8.5	29 (22.7)	29.6 ± 4.6	52 weeks
	Placebo		126	63.4 ± 10.2	30 (23.8)	29.3 ± 4.7	52 weeks
Gantz et al., 2017 [32]	Omarigliptin	25 mg qw, po	2100	63.7 ± 8.5	639 (30.4)	31.2 ± 5.5	96.1 weeks
	Placebo		2102	63.6 ± 8.5	615 (29.3)	31.4 ± 5.6	95.6 weeks
Rosenstock et al., 2019 [20]	Linagliptin	5 mg, qd, po	3494	66.1 ± 9.1	1346 (38.5)	31.4 ± 5.3	2.2 years
	Placebo		3485	65.6 ± 9.1	1243 (35.7)	31.3 ± 5.4	2.2 years
Hernandez et al., 2018 [23]	Albiglutide	30–50 mg, qw, IH	4731	64·1 ± 8·7	1427 (30.0)	32.3 ± 5.9	1.6 years
	Placebo		4732	64·2 ± 8·7	1467 (31.0)	32.3 ± 5.9	1.6 years
Marso et al., 2016 [24]	Liraglutide	1.8 mg, qd, IH	4668	64.2 ± 7.2	1657 (35.5)	32.5 ± 6.3	3.8 years
	Placebo		4672	64.4 ± 7.2	1680 (36.0)	32.5 ± 6.3	3.8 years
Gerstein et al., 2021 [33]	Efpeglenatide	4, 6 mg, qw, IH	2717	64.6 ± 8.2	925 (34.0)	32.9 ± 6.2	1.81 years
	Placebo		1359	64.4 ± 8.3	419 (30.8)	32.4 ± 6.0	1.81 years
Gerstein et al., 2019 [25]	Dulaglutide	1.5 mg, qw, IH	4949	66·2 ± 6·5	2306 (46·6)	32.3 ± 5.7	5·4 years
	Placebo		4952	66·2 ± 6·5	2283 (46·1)	32.3 ± 5.8	5·4 years
Husain et al., 2019 [26]	Semaglutide	14 mg, qd, po	1591	66.0 ± 7.0	507 (31.9)	32.3 ± 6.6	15.9 months
	Placebo		1592	66.0 ± 7.0	500 (31.4)	32.3 ± 6.4	15.9 months
Pfeffer et al., 2015 [35]	Lixisenatide	10–20 μg, qd, IH	3034	59.9 ± 9.7	923 (30.4)	30.1 ± 5.6	25 months
	Placebo		3034	60.6 ± 9.6	938 (30.9)	30.2 ± 5.8	25 months
Ruff et al., 2021 [36]	Exenatide	20, 60 mcg, qd, IH	2075	63 ± 2.5	778 (37.5)	32.4 ± 1.95	16 months
	Placebo		2081	63 ± 2.75	747 (35.9)	31.9 ± 1.875	16 months
Marso et al., 2016 [34]	Semaglutide	0.5 mg, qw, IH	826	64.6 ± 7.3	331 (40.1)	32.7 ± 6.29	2.1 years
	Semaglutide	1.0 mg, qw, IH	822	64.7 ± 7.1	304 (37.0)	32.9 ± 6.18	2.1 years
	Placebo	0.5 mg, qw, IH	824	64.8 ± 7.6	342 (41.5)	32.9 ± 6.35	2.1 years
	Placebo	1.0 mg, qw, IH	825	64.4 ± 7.5	318 (38.5)	32.7 ± 5.97	2.1 years
Holman et al., 2017 [37]	Exenatide	2 mg, qw, IH	7356	62.0 ± 3	2794 (38.0)	31.8 ± 2	3.2 years
	Placebo		7396	62.0 ± 3	2809 (38.0)	31.7 ± 1.975	3.2 years
Cannon et al., 2020 [38]	Ertugliflozin	5, 15 mg, qd, po	5499	64.4 ± 8.1	1633 (29.7)	31.9 ± 5.4	3.5 years
	Placebo		2747	64.4 ± 8.0	844 (30.7)	32.0 ± 5.5	3.5 years
Neal et al., 2017 [30]	Canagliflozin	100, 300 mg, qd, po	5795	63.2 ± 8.3	2036 (35.1)	31.9 ± 5.9	126.1 weeks
	Placebo		4347	63.4 ± 8.2	1597 (36.7)	32.0 ± 6.0	126.1 weeks
Wiviott et al., 2019 [28]	Dapagliflozin	10 mg, qd, po	8582	63.9 ± 6.8	3171 (36.9)	32.1 ± 6.0	4.2 years
	Placebo		8578	64.0 ± 6.8	3251 (37.9)	32.0 ± 6.1	4.2 years
Petrie et al., 2020 [40]	Dapagliflozin	10 mg, qd, po	1075	66.3 ± 9.9	240 (22.3)	29.3 ± 5.9	17.2 months
	Placebo		1064	66.7 ± 9.8	237 (22.3)	29.4 ± 6.1	16.6 months
Zinman, Lachin, and Inzucchi, 2015 [41]	Empagliflozin	10 mg, qd, po	2345	63.0 ± 8.6	692 (29.5)	30.6 ± 5.2	3.2 years
	Empagliflozin	25 mg, qd, po	2342	63.2 ± 8.6	659 (28.1)	30.6 ± 5.3	3.2 years
	Placebo		2333	63.2 ± 8.8	653 (28.0)	30.7 ± 5.2	3.1 years
Szarek et al., 2021 [43]	Sotagliflozin	200–400 mg, qd, po	608	69 ± 3.25	198 (32.6)	30.4 ± 2.0	9.0 months
	Placebo		614	70 ± 3.0	214 (34.9)	31.1 ± 1.8	9.0 months
Bhatt et al., 2020 [44]	Sotagliflozin	200–400 mg, qd, po	5292	69 ± 2.75	2347 (44.3)	31.9 ± 2.025	16.0 months
	Placebo		5292	69 ± 2.75	2407 (45.5)	31.7 ± 2.025	15.9 months
Anker et al., 2021 [42]	Empagliflozin	10 mg, qd, po	927	66.8 ± 10.0	210 (22.7)	28.8 ± 5.5	16 months
	Placebo		929	66.6 ± 10.3	218 (23.5)	28.6 ± 5.4	16 months
Mahaffey et al., 2019 [39]	Canagliflozin	100 mg, qd, po	1113	64.6 ± 8.2	357 (32.1)	31.6 ± 6.0	2.62 years
	Placebo		1107	64.6 ± 8.9	338 (30.5)	31.6 ± 6.1	2.62 years

*Note:* Data for age and constitution index were presented using mean ± standard deviation (Mean ± SD). Qd: once daily. bid: twice daily. qw: once weekly. po: oral. IH: subcutaneous injection; BMI: constitution index. Data for diabetes duration and HbA 1 c levels were expressed using the mean ± standard deviation (Mean ± SD). NA: No relevant data were available.

**TABLE 2:** Basic characteristics of the included literature.

Study	Intervention	Duration of diabetes mellitus (year)	HbA1c (%)	Coronary artery disease (*n*, %)	Hypertension (*n*, %)	Myocardial infarction (*n*, %)	Heart failure (*n*, %)	Cardiovascular diseases (*n*, %)	Stoke (*n*, %)	Peripheral vascular disease (*n*, %)
Scirica et al., 2013 [21]	Saxagliptin	10.3 ± 2.9	8.0 ± 1.4	6494 (78.4)	6725 (81.2)	3147 (38.0)	1056 (12.8)	NA	NA	NA
	Placebo	10.3 ± 2.8	8.0 ± 1.4	6465 (78.7)	6767 (82.4)	3090 (37.6)	1049 (12.8)	NA	NA	NA
Green et al., 2015 [18]	Sitagliptin	11.6 ± 8.1	7.2 ± 0.5	NA	NA	3133 (42.7)	1303 (17.8)	5397 (73.6)	NA	1217 (16.6)
	Placebo	11.6 ± 8.1	7.2 ± 0.5	NA	NA	3122 (42.5)	1340 (18.3)	5466 (74.5)	NA	1216 (16.6)
White et al., 2013 [19]	Alogliptin	7.1 ± 2.8	8.0 ± 1.1	NA	2229 (82.5)	2389 (88.4)	757 (28.0)	NA	195 (7.2)	262 (9.7)
	Placebo	7.3 ± 2.7	8.0 ± 1.1	NA	2240 (83.6)	2345 (87.5)	744 (27.8)	NA	193 (7.2)	252 (9.4)
McMurray et al., 2018 [31]	Vildagliptin	9.5 ± 8.1	7.8 ± 1.0	NA	112 (87.5)	82 (64.1)	128 (100.0)	NA	12 (9.4)	NA
	Placebo	9.1 ± 7.8	7.8 ± 1.1	NA	108 (85.7)	80 (63.5)	126 (100.0)	NA	11 (8.7)	NA
Gantz et al., 2017 [32]	Omarigliptin	12.0 ± 7.6	8.0 ± 0.9	NA	1998 (95.1)	NA	341 (16.2)	NA	NA	NA
	Placebo	12.1 ± 8.0	8.0 ± 0.9	NA	2010 (95.6)	NA	300 (14.3)	NA	NA	NA
Rosenstock et al., 2019 [20]	Linagliptin	15.0 ± 9.6	7.9 ± 1.0	NA	3171 (90.8)	NA	952 (27.2)	NA	NA	NA
	Placebo	14.5 ± 9.3	8.0 ± 1.0	NA	3178 (91.2)	NA	921 (26.4)	NA	NA	NA
Hernandez et al., 2018 [23]	Albiglutide	14.1 ± 8.6	8.76 ± 1.5	3333 (70)	4089 (86)	2223 (47)	954 (20)	NA	827 (17)	1195 (25)
	Placebo	14.2 ± 8.9	8.72 ± 1.5	3345 (71)	4095 (87)	2236 (47)	968 (20)	NA	854 (18)	1159 (24)
Marso et al., 2016 [24]	Liraglutide	12.8 ± 8.0	8.7 ± 1.6	NA	NA	1464 (31.4)	835 (17.9)	3831 (82.1)	NA	NA
	Placebo	12.9 ± 8.1	8.7 ± 1.5	NA	NA	1400 (30.0)	832 (17.8)	3767 (80.6)	NA	NA
Gerstein et al., 2021 [33]	Efpeglenatide	15.6 ± 8.8	8.90 ± 1.5	NA	2484 (91.4)	NA	487 (17.9)	2420 (89.1)	NA	NA
	Placebo	15.1 ± 8.7	8.94 ± 1.5	NA	1238 (91.1)	NA	250 (18.4)	1230 (90.5)	NA	NA
Gerstein et al., 2019 [25]	Dulaglutide	10.5 ± 7.3	7.3 ± 1.1	NA	4605 (93·0)	1028 (20·8)	421 (8·5)	1560 (31·5)	NA	NA
	Placebo	10.6 ± 7.2	7.4 ± 1.1	NA	4619 (93·3)	1007 (20·3)	432 (8·7)	1554 (31·4)	NA	NA
Mansoor Husain et al., 2019 [26]	Semaglutide	14.7 ± 8.5	8.2 ± 1.6	NA	NA	NA	NA	1350 (84.9)	NA	NA
	Placebo	15.1 ± 8.5	8.2 ± 1.6	NA	NA	NA	NA	1345 (84.5)	NA	NA
Pfeffer et al., 2015 [35]	Lixisenatide	9.2 ± 8.2	7.6 ± 1.3	NA	2340 (77.1)	672 (22.1)	676 (22.3)	NA	188 (6.2)	229 (7.5)
	Placebo	9.4 ± 8.3	7.7 ± 1.3	NA	2295 (75.6)	672 (22.1)	682 (22.5)	NA	143 (4.7)	237 (7.8)
Ruff et al., 2021 [36]	Exenatide	10.4 ± 2.4	8.0 ± 0.5	1032 (49.7)	NA	545 (26.3)	325 (15.7)	1578 (76.0)	NA	441 (21.2)
	Placebo	10.2 ± 2.4	8.0 ± 0.5	1004 (48.2)	NA	525 (25.2)	343 (16.5)	1581 (76.0)	NA	442 (21.2)
Steven P. Marso, 2016 [34]	Semaglutide	14.3 ± 8.2	8.7 ± 1.4	NA	772 (93.5)	266 (32.2)	201 (24.3)	NA	117 (14.2)	NA
	Semaglutide	14.1 ± 8.2	8.7 ± 1.5	NA	771 (93.8)	264 (32.1)	180 (21.9)	NA	113 (13.7)	NA
	Placebo	14.0 ± 8.5	8.7 ± 1.5	NA	756 (91.7)	267 (32.4)	190 (23.1)	NA	123 (14.9)	NA
	Placebo	13.2 ± 7.4	8.7 ± 1.5	NA	760 (92.1)	275 (33.3)	206 (25.0)	NA	138 (16.7)	NA
Holman et al., 2017 [37]	Exenatide	12.0 ± 2.5	8.0 ± 0.4	3898 (53.0)	NA	NA	1161 (15.8)	5394 (73.3)	NA	1400 (19.0)
	Placebo	12.0 ± 2.8	8.0 ± 0.4	3896 (52.7)	NA	NA	1228 (16.6)	5388 (72.9)	NA	1400 (18.9)
Cannon et al., 2020 [38]	Ertugliflozin	12.9 ± 8.3	8.2 ± 1.0	4144 (75.4)	NA	2625 (47.7)	1286 (23.4)	NA	230 (13.9)	1029 (18.7)
	Placebo	13.1 ± 8.4	8.2 ± 0.9	2112 (76.9)	NA	1329 (48.4)	672 (24.5)	NA	261 (15.8)	512 (18.6)
Neal et al., 2017 [30]	Canagliflozin	13.5 ± 7.7	8.2 ± 0.9	3234 (55.8)	5188 (89.5)	NA	803 (13.9)	NA	NA	1176 (20.3)
	Placebo	13.7 ± 7.8	8.2 ± 0.9	2487 (57.2)	3937 (90.6)	NA	658 (15.1)	NA	NA	937 (21.6)
Wiviott et al., 2019 [28]	Dapagliflozin	11.0 ± 2.5	8.3 ± 1.2	3474 (40.5)	NA	NA	852 (9.9)	NA	NA	522 (6.1)
	Placebo	10.0 ± 2.5	8.3 ± 1.2	3500 (40.8)	NA	NA	872 (10.2)	NA	NA	503 (5.9)
Petrie et al., 2020 [40]	Dapagliflozin	7.4 ± 2.7	7.4 ± 1.5	NA	NA	NA	1075 (100.0)	NA	NA	NA
	Placebo	7.4 ± 2.7	7.4 ± 1.6	NA	NA	NA	1064 (100.0)	NA	NA	NA
Zinman, Lachin, and Inzucchi, 2015 [41]	Empagliflozin	NA	8.1 ± 0.9	1782 (76.0)	NA	1107 (47.2)	240 (10.2)	NA	535 (22.8)	465 (19.8)
	Empagliflozin	NA	8.1 ± 0.8	1763 (75.3)	NA	1083 (46.2)	222 (9.5)	NA	549 (23.4)	517 (22.1)
	Placebo	NA	8.1 ± 0.8	1763 (75.6)	NA	1083 (46.4)	244 (10.5)	NA	553 (23.7)	479 (20.5)
Szarek et al., 2021 [43]	Sotagliflozin	10.2 ± 3.0	7.1 ± 0.5	NA	NA	NA	608 (100.0)	NA	NA	NA
	Placebo	10.2 ± 2.9	7.2 ± 0.5	NA	NA	NA	614 (100.0)	NA	NA	NA
Bhatt et al., 2020 [44]	Sotagliflozin	NA	8.3 ± 0.4	NA	NA	1051 (19.9)	1640 (31.0)	NA	472 (8.9)	NA
	Placebo	NA	8.3 ± 0.5	NA	NA	1057 (20.0)	1643 (31.0)	NA	474 (9.0)	NA
Anker et al., 2021 [42]	Empagliflozin	NA	7.4 ± 1.6	NA	730 (78.7)	NA	927 (100.0)	NA	NA	NA
	Placebo	NA	7.4 ± 1.6	NA	729 (78.5)	NA	929 (100.0)	NA	NA	NA
Mahaffey et al., 2019 [39]	Canagliflozin	16.3 ± 8.9	8.3 ± 1.3	NA	1085 (97.5)	215 (19.3)	266 (23.9)	NA	225 (20.2)	NA
	Placebo	16.5 ± 8.7	8.3 ± 1.3	NA	1081 (97.7)	227 (20.5)	265 (23.9)	NA	233 (21.0)	NA

*Note:* Data for diabetes duration and HbA 1 c levels were expressed using the mean ± standard deviation (Mean ± SD). NA: No relevant data were available.

### 3.3 Literature Quality Evaluation

**FIGURE 2:**
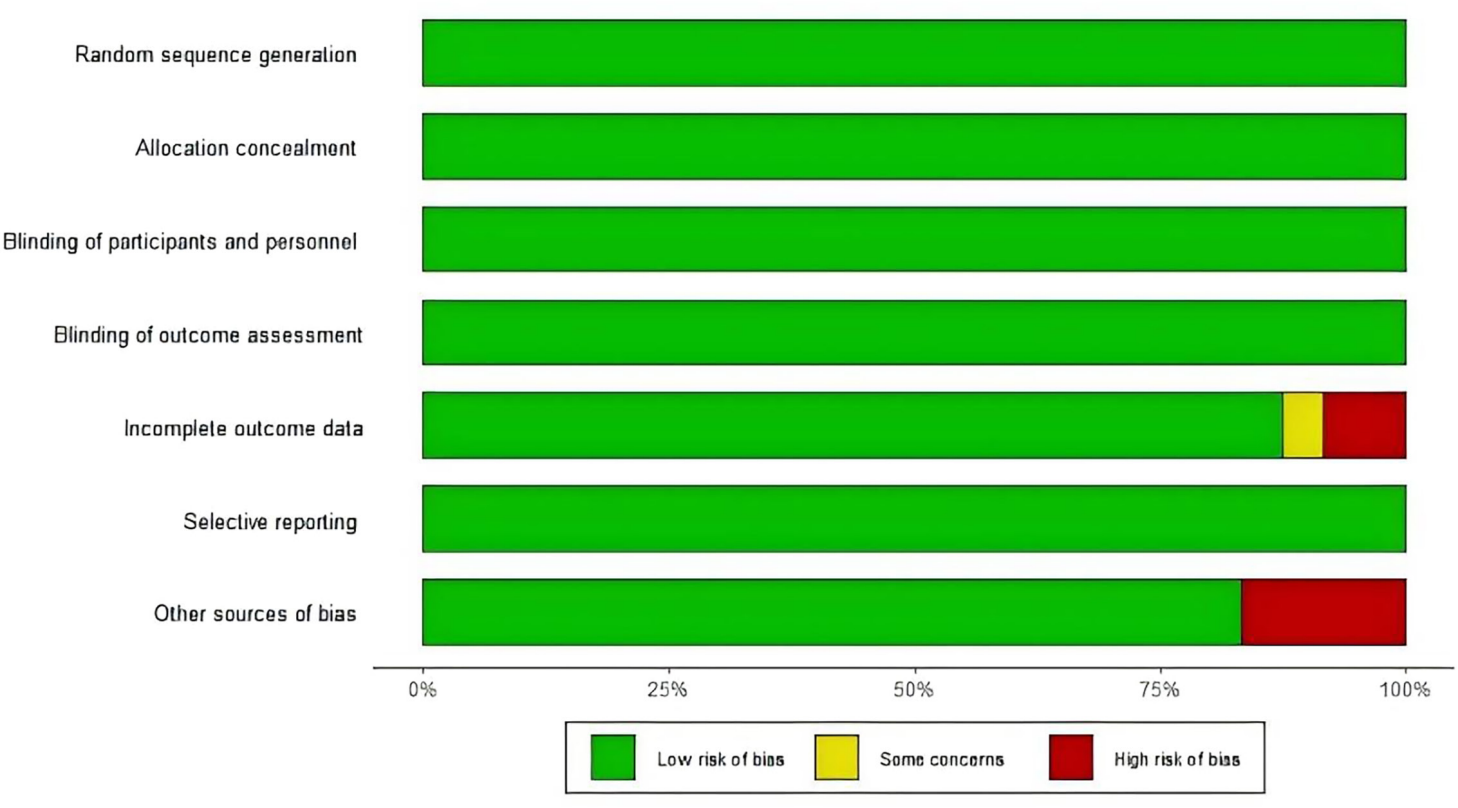
Risk of bias plot for literature quality evaluation.

The quality of the 24 included articles was assessed using the Cochrane Risk of bias assessment tool recommended by the Cochrane Collaboration. Twenty‐four RCTs were all multicenter, randomized, double‐blind, placebo‐controlled trials, and all described randomization and allocation concealment using an interactive voice/network response system. The overall literature quality was good, with 17 rated as low risk, 1 as unknown risk, and 6 as high risk. The specific quality evaluation situation is shown in Figure 2 and Figure S1.

### 3.4 Results of the Network Meta‐Analysis

#### 3.4.1 Mace

**FIGURE 3:**
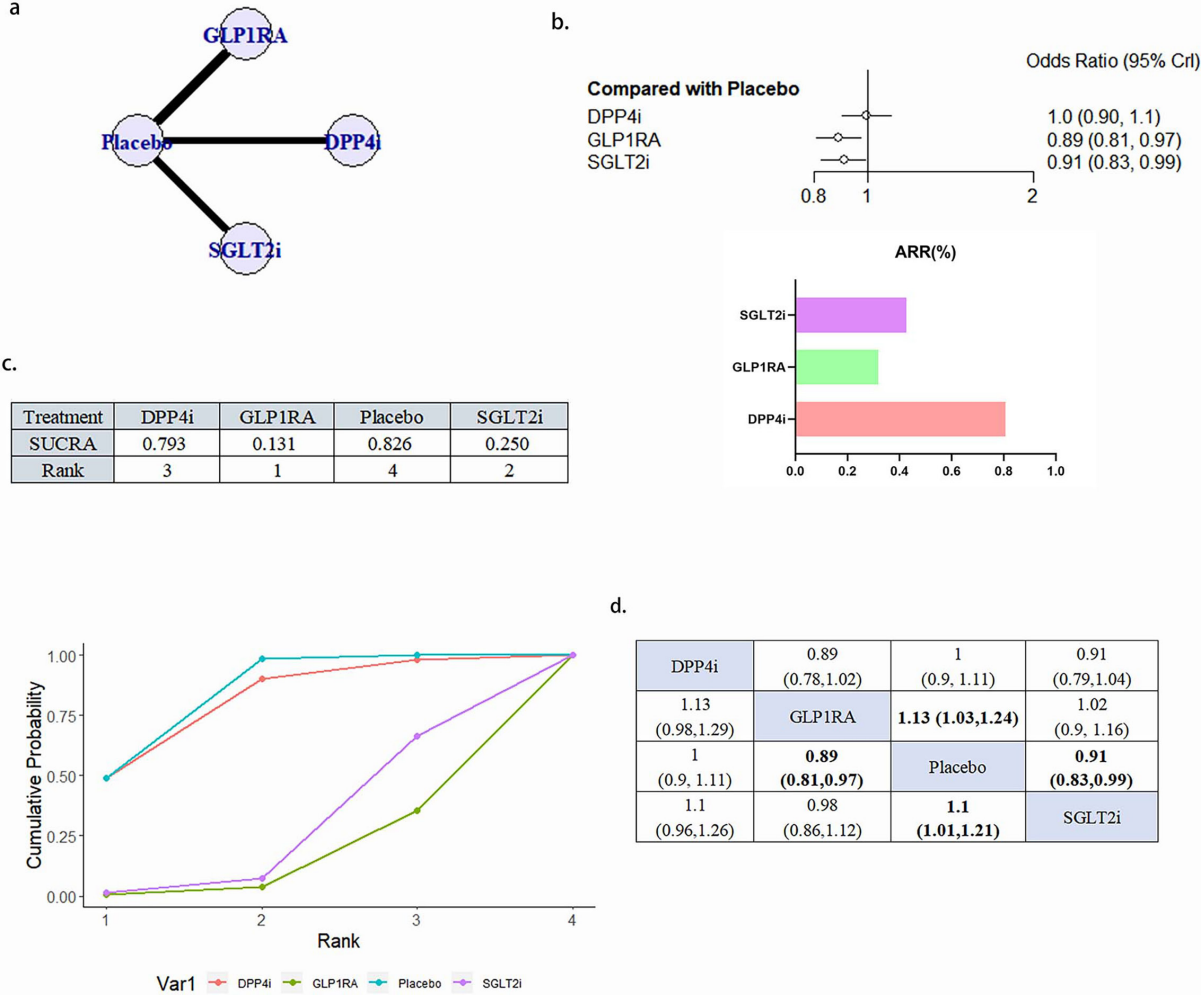
The effect of novel hypoglycemic drugs on the risk of MACE. (a) Network diagram. (b) Forest plot. (c) SUCRA and Line plots of the cumulative probabilities. (d) Pairwise comparison.

A total of 18 literature articles were included [19, 20, 21, 22, 24, 25, 27, 29, 30, 33, 35, 36, 37, 38, 39, 40, 43, 44, 45], 153 337 patients with T2DM and CVD participated in the study involving 4 interventions including 5 DPP4i related literature involving 23 899 patients; 7 GLP1RA related literature involving 25 103 patients; 6 SGLT2i related to 30 962 patients; 18 placebo related to 73 373 patients; network diagram as in Figure 3a. Compared with placebo, GLP1RA (OR 0.89, 95% CI 0.81–0.97) and SGLT2iOR (0.91, 95% CI 0.83–0.99) were statistically significant and GLP1RA ranked better than SGLT2i. DPP4i, although superior to placebo in the SUCRA ranking, did not show a statistically significant difference compared to placebo (Figure 3b–d). There was no publication bias (Figure S2a).

#### 3.4.2 Cardiovascular Death

**FIGURE 4:**
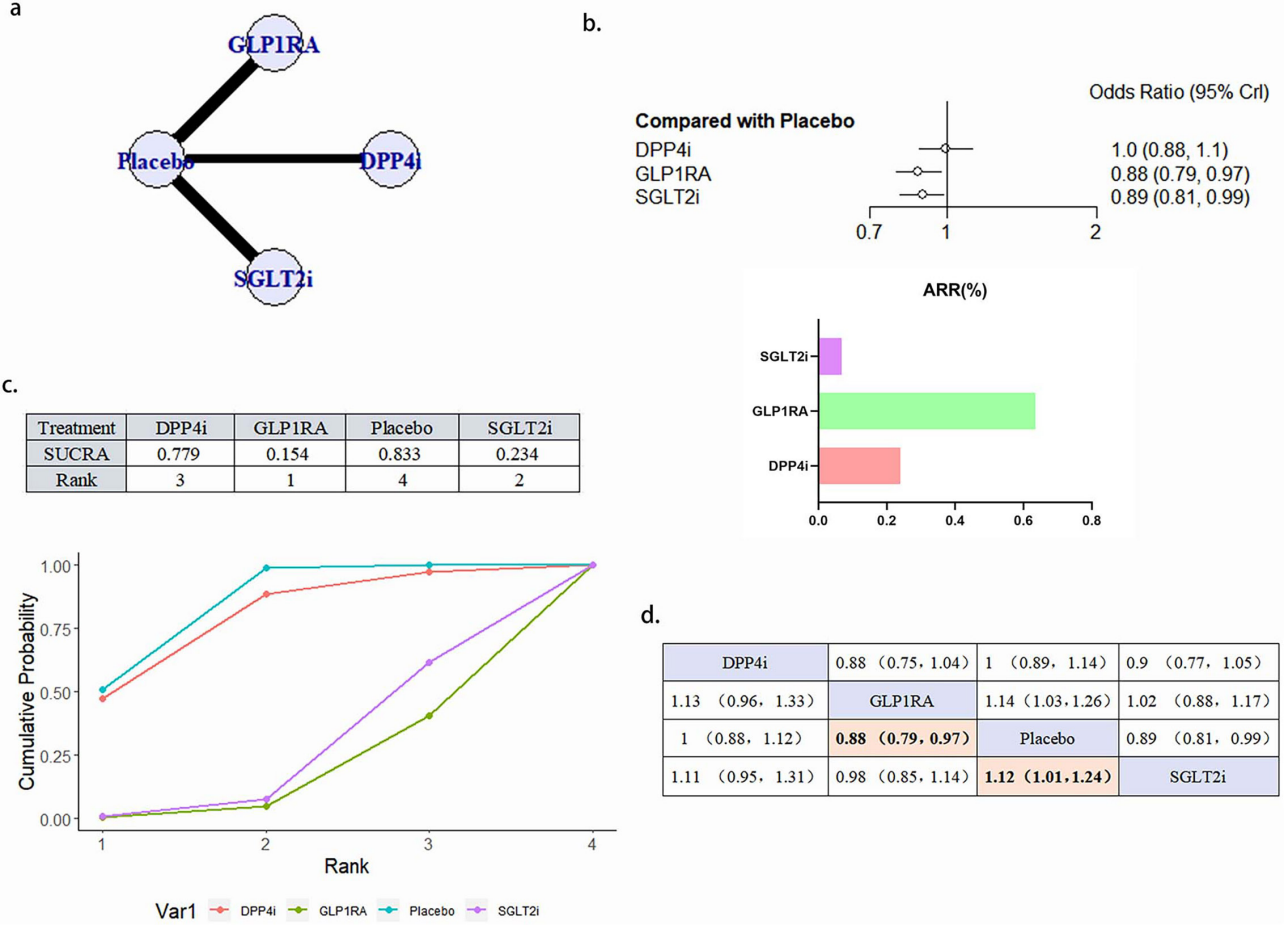
The effect of novel hypoglycemic drugs on the risk of Cardiovascular death. (a) Network diagram. (b) Forest plot. (c) SUCRA and Line plots of the cumulative probabilities. (d) Pairwise comparison.

A total of 24 literature articles were included [19, 20, 21, 22, 24, 25, 26, 27, 29, 30, 32, 33, 34, 35, 36, 37, 38, 39, 40, 41, 42, 43, 44, 45], 172 793 patients participated in the study involving 4 interventions, including 6 DPP4i related literature involving 24 027 patients; 9 GLP1RA related literature involving 32 769 patients; 9 SGLT2i related literature involving 34 192 patients; 24 placebo related literature, 81 805 patients; network diagram as in Figure 4a. Compared with placebo, GLP1RA (OR 0.88, 95% CI 0.79–0.97) and SGLT2i (OR 0.8995% CI 0.81–0.99) reduced the occurrence of deaths due to cardiovascular causes statistically, and GLP1RA ranked better than SGLT2i. DPP4i, although superior to placebo in the SUCRA ranking, did not show a statistically significant difference (Figure 4b–d). There was no publication bias (Figure S2b).

#### 3.4.3 Nonfatal Myocardial Infarction

**FIGURE 5:**
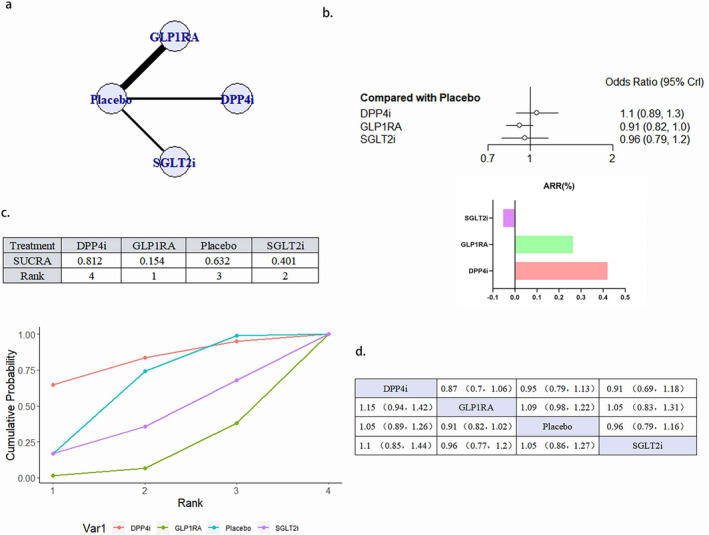
The effect of novel hypoglycemic drugs on the risk of nonfatal myocardial infarction. (a) Network diagram. (b) Forest plot. (c) SUCRA and Line plots of the cumulative probabilities. (d) Pairwise comparison.

A total of 15 literature articles were included [19, 20, 21, 24, 25, 26, 27, 34, 35, 36, 37, 38, 39, 40, 43], 108 752 patients participated in the study involving 4 interventions, including 3 DPP4i related articles, 13 527 patients participated in the study; GLP1RA related 932 768 patients participated in the study; 3 SGLT2i related literature, 11 299 patients participated in the study; and the placebo related articles 15, involving 51 157 patients; their network diagram as in Figure 5a. The magnitude of the four interventions in reducing nonfatal myocardial infarction was GLP1RA > SGLT2i > placebo > DPP4i, which was not statistically different (Figure 5b–d). There was no publication bias (Figure S2c).

#### 3.4.4 Nonfatal Stroke

**FIGURE 6:**
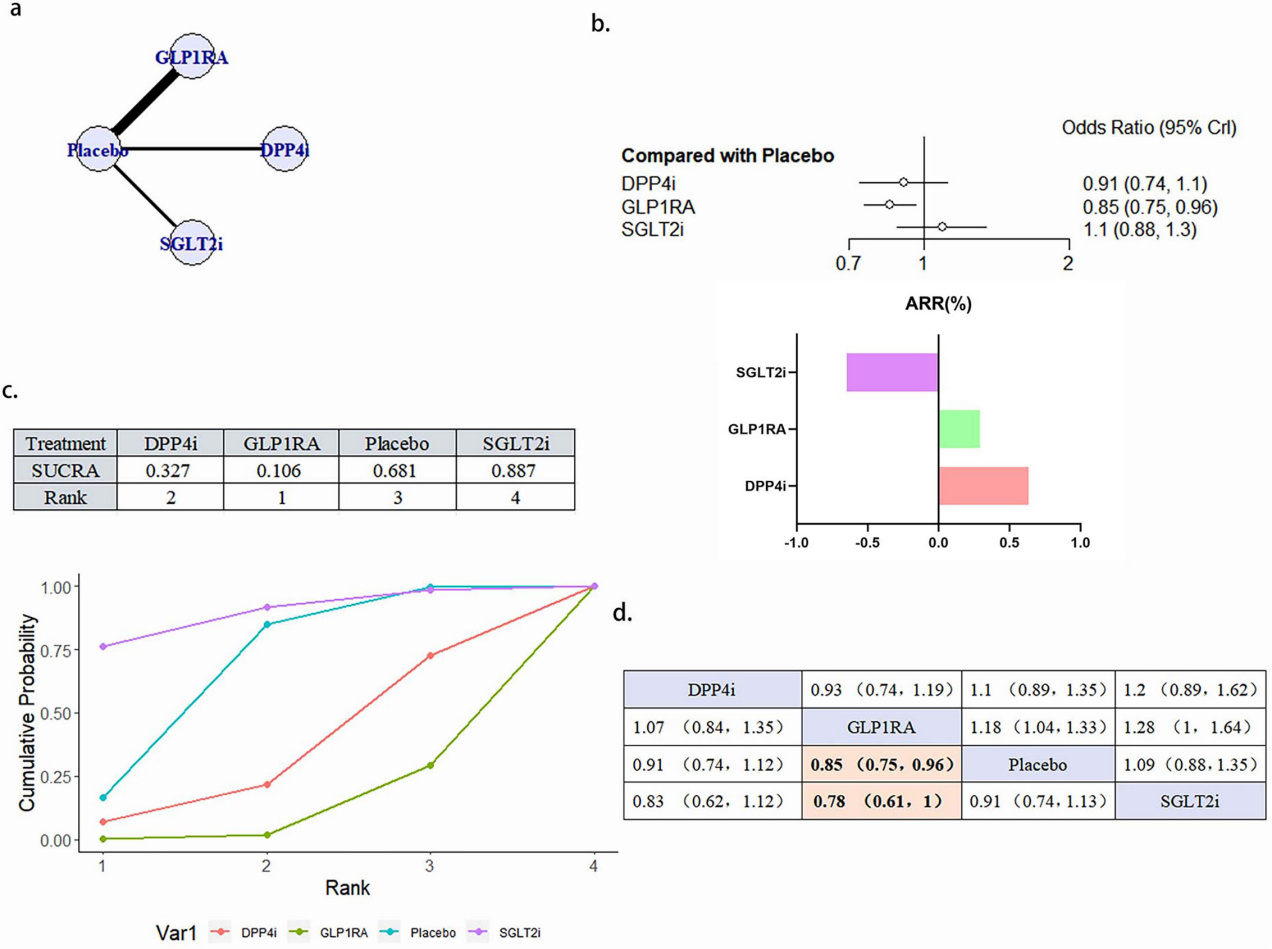
The effect of novel hypoglycemic drugs on the risk of nonfatal stoke. (a) Network diagram. (b) Forest plot. (c) SUCRA and Line plots of the cumulative probabilities. (d) Pairwise comparison.

A total of 15 literature articles were included [19, 20, 21, 24, 25, 26, 27, 34, 35, 36, 37, 38, 39, 40, 43], 108 752 patients participated in the study involving 4 interventions, including 3 DPP4i related literature, 13 527 patients participated in the study; GLP1RA related literature 932 768 patients participated in the study; 3 SGLT2i related literature, 11 299 patients participated in the study; and the placebo related 15, involving 51 157 patients; their network diagram as in Figure 6a. The GLP1RA (OR 0.85, 95% CI 0.75–0.96) reduced the occurrence of nonfatal stroke compared with placebo. The ranking of the four interventions in reducing the risk of nonfatal stroke was GLP1RA > DPP4i > placebo > SGLT2i. Although DPP4i ranking was better than placebo, the comparison results were not statistically significant (Figure 6b–d). There was no publication bias (Figure S2d).

#### 3.4.5 HHF

**FIGURE 7:**
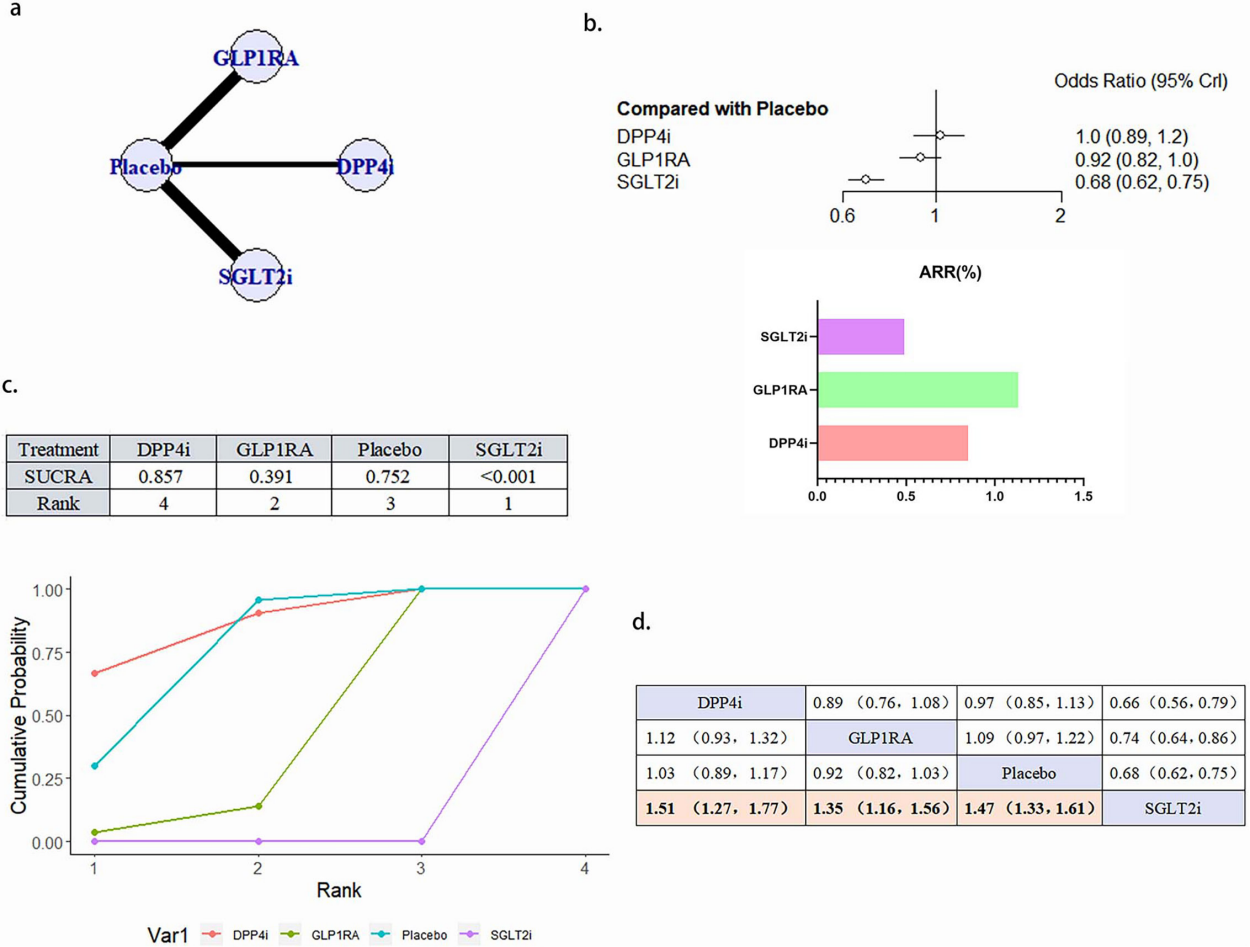
The effect of novel hypoglycemic drugs on the risk of HHF. (a) Network diagram. (b) Forest plot. (c) SUCRA and Line plots of the cumulative probabilities. (d) Pairwise comparison.

A total of 21 literature articles were included [19, 21, 22, 25, 26, 27, 29, 30, 33, 34, 35, 36, 37, 38, 39, 40, 41, 42, 43, 44, 45], 157 696 patients participated in the study, involving 4 interventions, including 4 literature related to DPP4i, 21 198 patients participated in the study; GLP1RA 8‐related literature, 28 038 patients participated in the study; 9 SGLT2i related literature, 33 578 patients participated in the study; 21 placebo related, 74 482 patients participated in the study; their network diagram as in Figure 7a. SGLT2i (OR 0.68, 95% CI 0.62–0.75) can reduce the occurrence of HHF in patients and is a protective factor. The ability of the four interventions to reduce the risk of HHF was SGLT2i > GLP1RA > placebo > DPP4i, and the results between SGLT2i and GLP1RA, placebo and DPP4i were statistically different (Figure 7b–d). There was no publication bias (Figure S2e).

#### 3.4.6 All‐Cause Mortality

**FIGURE 8:**
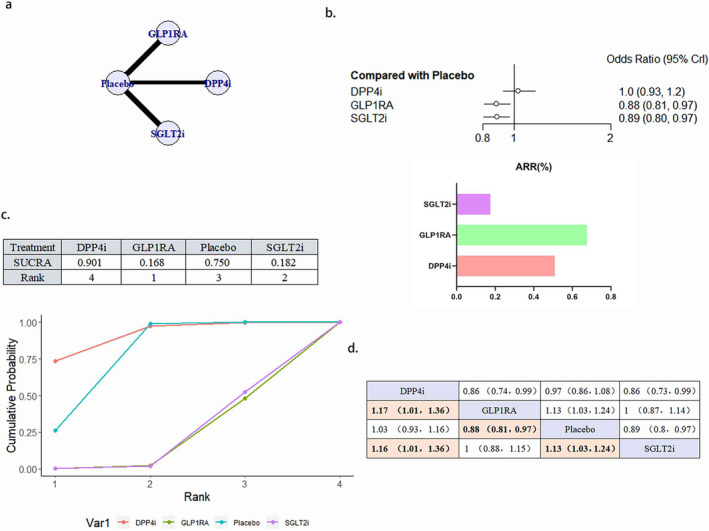
The effect of novel hypoglycemic drugs on all‐cause mortality (a) Network diagram. (b) Forest plot. (c) SUCRA and Line plots of the cumulative probabilities. (d) Pairwise comparison.

A total of 23 literature articles were included [19, 20, 21, 22, 24, 25, 26, 27, 29, 30, 32, 33, 34, 35, 36, 37, 38, 39, 40, 41, 43, 44, 45], 170 937 patients participated in the study involving 4 interventions, including 6 articles concerning DPP4i involving 24 047 patients; 9 GLP1RA related articles involving 32 769 patients; 8 SGLT2i related articles involving 32 651 patients; 23 placebo related articles involving 81 490 patients; and network diagram as in Figure 8a. GLP1RA (OR 0.88, 95% CI 0.81–0.97) and SGLT2i (OR 0.89, 95% CI 0.80–0.97) reduced the occurrence of all‐cause death as a protective factor. The merits of the four interventions in reducing the risk of all‐cause death was GLP1RA > SGLT2i > placebo > DPP4i. No significant difference between the effects of GLP1RA and SGLT2i (Figure 8b–d). There was no publication bias (Figure S2f).

## 4 Discussion

### 4.1 Diabetes Mellitus and Cardiovascular Disease

The prevalence of diabetes continues to be elevated worldwide, and T2DM increases the risk of concurrent CVD, which seriously affects the quality of life and prognosis of patients. The CVD associated with T2DM mainly includes ischemic heart disease, HF, stroke, coronary artery disease, and peripheral artery disease, and at least half of the deaths in patients with T2DM are attributed to these complications [45]. Therefore, CVD has received great attention in the disease progression and prognosis of T2DM. The mechanism of T2DM complicated by CVD is mainly based on hyperglycemia and insulin resistance, the associated abnormal lipid metabolism in the majority of patients, and a series of secondary pathophysiological changes [46]. Insulin resistance may lead to an elevated relative risk of cardiovascular events [47, 48], and sustained increases in blood glucose can lead to macrovascular and microvascular complications in patients with T2DM [47, 48], excessive accumulation of lipids may lead to cardiac insulin resistance, fibrosis, and diastolic dysfunction [49].

Current guidelines recommend that all patients with diabetes mellitus require regular assessment of cardiovascular risk. Similarly, patients with CVD must be screened for diabetes early after diagnosis and maintain good glucose management, assessing the 10‐year cardiovascular risk using the new SCORE2‐Diabetes score in patients with established diabetes but no confirmed atherosclerotic CVD or no serious target organ damage [3]. Focusing on the prevention of cardiovascular events in high‐risk populations may reduce mortality and reduce the economic burden [3].

Although numerous animal experiments have proved the protective effect of new hypoglycemic drugs on cardiovascular and cerebrovascular vessels. However, there are still some differences between clinical trials and meta‐analysis regarding its effect on cardiovascular outcomes. This study comprehensively analyzed multiple large, multicenter, long‐term follow‐up RCTs on the effects of novel hypoglycemic drugs on cardiovascular outcome, using the network meta‐analysis method to achieve indirect comparison of new glucose‐lowering drugs, and we provided some evidence‐based basis for the long‐term cardiovascular benefit of new glucose‐lowering drugs in T2D patients with CVD.

### 4.2 Cardiovascular Benefits of New Hypoglycemic Agents

The analysis of MACE, cardiovascular death and all‐cause deaths showed superior GLP1RA and SGLT2i over placebo, while DPP4i did not show a statistical difference from placebo. There was no significant difference in the efficacy of GLP1RA and SGLT2i in improving MACE, cardiovascular death and all‐cause death in T2DM patients with CVD, which is consistent with the existing analysis [50].

In the analysis of nonfatal MI events, DPP4i, GLP1RA, and SGLT2i showed superior effects to placebo, but were noninferior to placebo, consistent with the results of the existing meta‐analysis [51]. In this study, DPP4i did not show associated with a reduced risk of nonfatal MI, but in animal experiments, DPP4i was found to act on SDF‐1 α (stromal cell‐derived factor‐1alpha) to exert anti‐inflammatory and stable plaque effects, and reduce infarct area in diabetic rats [52]. The same happened with GLP1RA, although it was not associated with reducing the risk of nonfatal myocardial infarction in this study, GLP1RA was found to reduce the area of myocardial infarction and improve cardiac function by inhibiting myocardial Ca^2+^ overload and cardiomyocyte apoptosis. The pathophysiological changes of MI are very complex, and the role of GLP1RA on MI needs to be further verified.

In animal experiments, it was found that SGLT2i may improve myocardial infarction by some mechanisms, such as downregulating autophagy flux [53], inhibiting inflammatory [54, 55], inhibiting oxidative stress [56, 57], improving energy metabolism [58, 59], increasing ketogenesis, reducing cardiac fatty acid oxidation and reducing cardiac oxygen consumption [60, 61], and improving endothelial dysfunction [62]. The EMPDY test revealed that early use of Empagliflozin resulted in decreased expression of sudden cardiac death markers [63]. A study of 583 patients with acute myocardial infarction found a significant reduction of SGLT2i with small infarct size [64]. From animal experiment [65, 66] to clinical trials, SGLT2i was found to benefit the reduction of infarct size, improvement of left ventricular function and delay of ventricular remodeling, but in this study, SGLT2i did not reduce the risk of nonfatal myocardial infarction in T2DM patients with CVD, which is consistent with the results of existing meta‐analysis [67, 68]. Therefore, some scholars speculate that the cardiovascular protective effect of SGLT2i does not reduce the risk of acute myocardial infarction, but improves the myocardial injury induced by myocardial infarction through direct cytoprotective effect, so as to reduce the progression of ischemic cardiomyopathy and HF, thus improving the immediate and long‐term survival rates after acute myocardial infarction [69]. Further basic and clinical studies are still needed to confirm the potential of SGLT2i for the treatment of acute myocardial infarction.

In the analysis of nonfatal stroke events, it was found that only GLP1RA reduced the risk of nonfatal stroke better than placebo and DPP4i, while SGLT2i seemed to exacerbate the occurrence of nonfatal stroke. Diabetes is an independent risk factor for stroke and the risk of stroke is twice that in nondiabetic patients [70, 71] Diabetes mellitus is also a risk factor for recurrent stroke, with a higher risk of adverse outcomes after stroke [72, 73]. Therefore, in the 2008 guidance, FDA (Food and Drug Administration) referred to the need for new drugs to improve glycemic control in T2DM patients for cardiovascular outcome testing to assess the cardiovascular safety of new drugs, and one of the important indicators is stroke. It was found that GLP1RA has a neuroprotective effect independent of glycemic control, which attenuate oxidative stress, apoptosis and the production of pro‐inflammatory factors by affecting signaling pathways, which may effectively reduce the risk of stroke [74, 75]. Although the mechanism of action of DPP4i is similar to that of GLP1RA, DPP‐4 does not cross the BBB and does not induce plasma GLP‐1 elevation, which may be why DPP4i does not show a beneficial effect on stroke [74, 75].

In animal studies, SGLT2i was found to reduce neuronal damage and achieve neuroprotection through inhibition of brain oxidative stress, inflammatory, and apoptotic markers [76, 77]. However, SGLT2i did not show the beneficial effect on stroke in the clinical application. EMPA‐REG OUTCOME is the first study to report that Empagliflozin may be associated with an elevated risk of stroke [29]. Although this elevated risk did not reach a statistical difference, it still attracts much attention. Further studies found an increased hematocrit in Empagliflozin‐treated patients compared to placebo‐treated patients [78]. And a high hematocrit was associated with an increased risk of stroke [79], and positively associated with reperfusion and reduced infarct size after ischemic stroke [80, 81]. Studies have found that the glucose reduction, weight loss and antihypertensive effect of SGLT2i can reduce the risk of stroke, but at the same time, SGLT2i may lead to hypovolemia and hypotension, leading to increased risk of ischemic stroke and increased risk of ischemic stroke [82]. In a meta‐analysis assessing the effect of SGLT2i on stroke subtypes, SGLT2i found no obvious effect on the total risk of stroke, the risk of nonfatal stroke and only a potential protection against hemorrhagic stroke, probably attributed to the hypolipidemic effect of SGLT2i [83]. This also suggests that our analysis of the effect of SGLT2i on stroke requires a more carefully differentiated subtypes. In clinical application, attention should still be paid to the excavation of patients' previous medical history. The application of SGLT2i in patients with higher hematocrit may aggravate the risk of stroke.

In the analysis of HHF events, SGLT2i was found to significantly reduce the occurrence of HHF events, while DPP4i and GLP1RA did not show an effect of reducing HHF risk. Diabetes mellitus is an important risk factor for HF [84]. The prevalence of HF in diabetic patients is as high as 22%, and the incidence is increasing [85]. Compared with nondiabetic patients, diabetic patients have a 2‐ to 4‐fold increased risk of HF, and HF occurs earlier in patients with T2DM. Diabetic patients with HF are also at more than 50% higher risk of cardiovascular death than HF patients without diabetes. HF is the most common first‐time CVD manifestation in patients with T2DM [86, 87, 88]. The SGLT2i was found to have a diuretic effect and long‐term application results in decreased systolic blood pressure and sustained reductions in body weight and plasma volume [89], and less impact on blood volume, arterial filling and organ perfusion than general diuretics [90]. SGLT2i Also by reducing cardiac hypertrophy and myopathy development and progression and improving myocardial energy metabolism [91, 92]. Furthermore, SGLT2i may improve renal outcomes and may reduce the burden of cardiorenal syndrome in patients with HF [93]. The analysis found that the effect of SGLT2i on reducing the risk of HF hospital admission was very significant [94], and the beneficial effect of SGLT2i on HF was independent of glycemic control [95, 96]. Therefore, subsequent clinical trials were conducted to evaluate the cardiovascular benefits of SGLT2i in patients with HF (with or without diabetes), and the experimental results further supported the beneficial effects of SGLT2i on HF [97, 98]. Therefore, the European Society of Cardiology guidelines starting in 2021 recommend adding SGLT2i to HF treatment options in patients with HFrEF (Heart failure with reduced ejection fraction) with reduced ejection fraction [99]. The 2022 Heart failure guidelines issued by the Acardiology/American Heart Association/Heart Failure Society recommend the use of SGLT2i in patients with HFrEF, and also recommend SGLT2i in heart failure patients with HFrEF [100].

### 4.3 Limitations

First, the number of included studies was small, and the included studies were placebo‐controlled trials. Therefore, there are only indirect comparisons between the new hypoglycemic drugs, which cannot explore the consistency between direct comparison and indirect comparison results, and high‐quality head‐to‐head direct comparison studies are needed in the future.

Second, the study subjects were patients with T2DM and CVD, but the types of CVD patients at baseline were different, and the proportion of each disease varied greatly, which may have an influence on the results. There were differences in basal hypoglycemic, hypolipidemic, and cardioprotective medication regimen when patients were enrolled in the study. In addition, there is no differentiation of drug dose and administration method in this experiment, which may also lead to the reasons of the heterogeneity between studies.

Finally, a large number of literature reported the number of stroke and nonfatal stroke, and did not distinguish stroke subtypes, so it is impossible to explore whether the effect of SGLT2i on stroke is limited to a certain stroke subtype.

## 5 Conclusion

This network meta‐analysis of 24 RCTs demonstrated that, GLP1RA and SGLT2i reduced MACE, cardiovascular mortality and all‐cause mortality in T2DM patients with CVD, with no significant difference in efficacy, and DPP4i was noninferior to placebo. Only GLP1RA reduced the risk of nonfatal stroke, and only SGLT2i reduced the risk of HHF. Both GLP1RA, SGLT2i, and DPP4i had no significant beneficial effects on the risk of nonfatal myocardial infarction.

## Author Contributions

Yan Dai participated in the topic selection and revised the article. Saixian Shi and Xiaofeng Li participated in the topic selection, article writing, illustrations, and revised the article. Ye Chen and Jiahao Li participated in the topic selection, article modification and put forward many valuable guidelines.

## Disclosure

All authors have participated in the work and have reviewed and agreed with the final version of the manuscript. All authors consent for publication this paper.

## Conflicts of Interest

The authors declare no conflicts of interest.

## Supporting information


**Table S1.** Search strategy.
**Figure S1.** Total plot of risk of bias for literature quality evaluation.
**Figure S2.** Comparison‐adjusted funnel plot.
