# Supplementary material for: Cardiovascular Therapy Benefits of Novel Antidiabetic Drugs in Patients With Type 2 Diabetes Mellitus Complicated With Cardiovascular Disease: A Network Meta‐Analysis
Source: J Diabetes. 2025 Jan 9;17(1):e70044. doi: 10.1111/1753-0407.70044 (PMC11717902; doi:10.1111/1753-0407.70044)
Supplement: Supplementary file 1 — Table S1. Search strategy. Figure S1. Total plot of risk of bias for literature quality evaluation. Figure S2. Comparison‐adjusted funnel plot. [file JDB-17-e70044-s001.doc]

**Supplementary Table 1. Search strategy**

**pubmed:**

| #1 | ((((((((((((((((((((((((((((((((((((((((((((((((((DPP 4 Inhibitor[Title/Abstract]) OR (DPP 4 Inhibitor[Title/Abstract])) OR (Gliptin[Title/Abstract])) OR (Dipeptidyl Peptidase 4 Inhibitor[Title/Abstract])) OR (Sitagliptin[Title/Abstract])) ) OR (Januvia[Title/Abstract])) OR (Linagliptin[Title/Abstract])) OR (Tradjenta[Title/Abstract])) OR (Vildagliptin[Title/Abstract])) OR (saxagliptin[Title/Abstract])) OR (alogliptin[Title/Abstract])) OR (LC15-0444[Title/Abstract])) OR (Gemigliptin[Title/Abstract])) OR (dutogliptin[Title/Abstract])) OR (anagliptin[Title/Abstract])) OR (evogliptin[Title/Abstract])) OR (gosogliptin[Title/Abstract])) OR (Omarigliptin[Title/Abstract])) OR (teneligliptin[Title/Abstract])) OR (trelagliptin[Title/Abstract])) OR (Glucagon-like peptide-1 receptor agonist[Title/Abstract])) OR (GLP-1 receptor agonist[Title/Abstract])) OR (GLP-1 RAs[Title/Abstract])) OR (incretin mimetic[Title/Abstract])) OR (semaglutide[Title/Abstract])) OR (Liraglutide[Title/Abstract])) OR (rGLP-1 protein[Title/Abstract])) OR (albiglutide[Title/Abstract])) OR (lixisenatide[Title/Abstract])) OR (Exenatide[Title/Abstract])) OR (Exendin 4[Title/Abstract])) OR (dulaglutide[Title/Abstract])) OR (taspoglutide[Title/Abstract])) OR (Sodium Glucose Transporter 2 Inhibitor[Title/Abstract])) OR (SGLT2 Inhibitor[Title/Abstract])) OR (Inhibitor, SGLT2[Title/Abstract])) OR (Gliflozin[Title/Abstract])) OR (SGLT 2 Inhibitor[Title/Abstract])) OR (Canagliflozin[Title/Abstract])) OR (dapagliflozin[Title/Abstract])) OR (empagliflozin[Title/Abstract])) OR (ertugliflozin[Title/Abstract])) OR (ipragliflozin[Title/Abstract])) OR (sotagliflozin[Title/Abstract])) OR (LX4211[Title/Abstract])) OR (tofogliflozin[Title/Abstract])) OR (luseogliflozin[Title/Abstract])) OR (remogliflozin[Title/Abstract])) OR (sergliflozin[Title/Abstract])) OR (("Dipeptidyl-Peptidase IV Inhibitors"[Mesh]) OR "Sodium-Glucose Transporter 2 Inhibitors"[Mesh]) | 26726 |
| --- | --- | --- |
| #2 | (((((((((((("Cardiovascular Diseases"[Mesh]) OR "Acute Coronary Syndrome"[Mesh]) OR "Heart Failure"[Mesh]) OR "Coronary Disease"[Mesh]) OR "Atherosclerosis"[Mesh]) OR "Arrhythmias, Cardiac"[Mesh]) OR "Atrial Fibrillation"[Mesh]) OR "Ventricular Fibrillation"[Mesh]) OR "Angina Pectoris"[Mesh]) OR "Myocardial Infarction"[Mesh]) OR "Hypertension"[Mesh]) OR ( "Cardiomyopathies"[Mesh] OR "Diabetic Cardiomyopathies"[Mesh] )) OR "Stroke"[Mesh] | 2748553 |
| #3 | (((((((((((((((((((((((((((((((((((((((Cardiovascular disease[Title/Abstract]) OR (CVD[Title/Abstract])) OR (ASCVD[Title/Abstract])) OR (Major Adverse Cardiac Events[Title/Abstract])) OR (Cardiac Event[Title/Abstract])) OR (Adverse Cardiac Event[Title/Abstract])) OR (Coronary Syndrome, Acute[Title/Abstract])) OR (Syndrome, Acute Coronary[Title/Abstract])) OR (Cardiac Failure[Title/Abstract])) OR (Heart Decompensation[Title/Abstract])) OR (Myocardial Failure[Title/Abstract])) OR (HF[Title/Abstract])) OR (CHF[Title/Abstract])) OR (HFpEF[Title/Abstract])) OR (HFrEF[Title/Abstract])) OR (Coronary Heart Disease[Title/Abstract])) OR (Atheroscleroses[Title/Abstract])) OR (Atherogenesis[Title/Abstract])) OR (Arrhythmia[Title/Abstract])) OR (Cardiac Dysrhythmia[Title/Abstract])) OR (Atrial Fibrillations[Title/Abstract])) OR (Fibrillation[Title/Abstract])) OR (Ventricular Fibrillations[Title/Abstract])) OR (Stenocardia[Title/Abstract])) OR (Angor[Title/Abstract])) OR (Infarction, Myocardial[Title/Abstract])) OR (Cardiovascular Stroke[Title/Abstract])) OR (Myocardial Infarct[Title/Abstract])) OR (Heart Attack[Title/Abstract])) OR (High Blood Pressure[Title/Abstract])) OR (Cardiomyopathy[Title/Abstract])) OR (Myocardiopathy[Title/Abstract])) OR (Myocardial Disease[Title/Abstract])) OR (Cerebrovascular Accident[Title/Abstract])) OR (Stokes[Title/Abstract])) OR (CVA[Title/Abstract])) OR (Cerebrovascular Apoplexy[Title/Abstract])) OR (Brain Vascular Accident[Title/Abstract])) OR (Cerebrovascular Stroke[Title/Abstract])) OR (Apoplexy[Title/Abstract]) | 645589 |
| 4=#2 OR #3 | ((((((((((((((((((((((((((((((((((((((((Cardiovascular disease[Title/Abstract]) OR (CVD[Title/Abstract])) OR (ASCVD[Title/Abstract])) OR (Major Adverse Cardiac Events[Title/Abstract])) OR (Cardiac Event[Title/Abstract])) OR (Adverse Cardiac Event[Title/Abstract])) OR (Coronary Syndrome, Acute[Title/Abstract])) OR (Syndrome, Acute Coronary[Title/Abstract])) OR (Cardiac Failure[Title/Abstract])) OR (Heart Decompensation[Title/Abstract])) OR (Myocardial Failure[Title/Abstract])) OR (HF[Title/Abstract])) OR (CHF[Title/Abstract])) OR (HFpEF[Title/Abstract])) OR (HFrEF[Title/Abstract])) OR (Coronary Heart Disease[Title/Abstract])) OR (Atheroscleroses[Title/Abstract])) OR (Atherogenesis[Title/Abstract])) OR (Arrhythmia[Title/Abstract])) OR (Cardiac Dysrhythmia[Title/Abstract])) OR (Atrial Fibrillations[Title/Abstract])) OR (Fibrillation[Title/Abstract])) OR (Ventricular Fibrillations[Title/Abstract])) OR (Stenocardia[Title/Abstract])) OR (Angor[Title/Abstract])) OR (Infarction, Myocardial[Title/Abstract])) OR (Cardiovascular Stroke[Title/Abstract])) OR (Myocardial Infarct[Title/Abstract])) OR (Heart Attack[Title/Abstract])) OR (High Blood Pressure[Title/Abstract])) OR (Cardiomyopathy[Title/Abstract])) OR (Myocardiopathy[Title/Abstract])) OR (Myocardial Disease[Title/Abstract])) OR (Cerebrovascular Accident[Title/Abstract])) OR (Stokes[Title/Abstract])) OR (CVA[Title/Abstract])) OR (Cerebrovascular Apoplexy[Title/Abstract])) OR (Brain Vascular Accident[Title/Abstract])) OR (Cerebrovascular Stroke[Title/Abstract])) OR (Apoplexy[Title/Abstract])) OR ((((((((((((("Cardiovascular Diseases"[Mesh]) OR "Acute Coronary Syndrome"[Mesh]) OR "Heart Failure"[Mesh]) OR "Coronary Disease"[Mesh]) OR "Atherosclerosis"[Mesh]) OR "Arrhythmias, Cardiac"[Mesh]) OR "Atrial Fibrillation"[Mesh]) OR "Ventricular Fibrillation"[Mesh]) OR "Angina Pectoris"[Mesh]) OR "Myocardial Infarction"[Mesh]) OR "Hypertension"[Mesh]) OR ( "Cardiomyopathies"[Mesh] OR "Diabetic Cardiomyopathies"[Mesh] )) OR "Stroke"[Mesh]) | 2964964 |
| #5 | (((((((((Diabetes Mellitus, Noninsulin-Dependent[Title/Abstract]) OR (Non-Insulin-Dependent Diabetes Mellitus[Title/Abstract])) OR (Diabetes Mellitus, Type II[Title/Abstract])) OR (NIDDM[Title/Abstract])) OR (T2DM[Title/Abstract])) OR (Type 2 Diabetes Mellitus[Title/Abstract])) OR (Noninsulin-Dependent Diabetes Mellitus[Title/Abstract])) OR (Type 2 Diabetes[Title/Abstract])) OR (Diabetes, Type 2[Title/Abstract])) OR ("Diabetes Mellitus, Type 2"[Mesh]) | 238910 |
| #6 = #1 AND #4 AND #5 | ((((((((((((((((((((((((((((((((((((((((((Cardiovascular disease[Title/Abstract]) OR (CVD[Title/Abstract])) OR (ASCVD[Title/Abstract])) OR (Major Adverse Cardiac Events[Title/Abstract])) OR (Cardiac Event[Title/Abstract])) OR (Adverse Cardiac Event[Title/Abstract])) OR (Coronary Syndrome, Acute[Title/Abstract])) OR (Syndrome, Acute Coronary[Title/Abstract])) OR (Cardiac Failure[Title/Abstract])) OR (Heart Decompensation[Title/Abstract])) OR (Myocardial Failure[Title/Abstract])) OR (HF[Title/Abstract])) OR (CHF[Title/Abstract])) OR (HFpEF[Title/Abstract])) OR (HFrEF[Title/Abstract])) OR (Coronary Heart Disease[Title/Abstract])) OR (Atheroscleroses[Title/Abstract])) OR (Atherogenesis[Title/Abstract])) OR (Arrhythmia[Title/Abstract])) OR (Cardiac Dysrhythmia[Title/Abstract])) OR (Atrial Fibrillations[Title/Abstract])) OR (Fibrillation[Title/Abstract])) OR (Ventricular Fibrillations[Title/Abstract])) OR (Stenocardia[Title/Abstract])) OR (Angor[Title/Abstract])) OR (Infarction, Myocardial[Title/Abstract])) OR (Cardiovascular Stroke[Title/Abstract])) OR (Myocardial Infarct[Title/Abstract])) OR (Heart Attack[Title/Abstract])) OR (High Blood Pressure[Title/Abstract])) OR (Cardiomyopathy[Title/Abstract])) OR (Myocardiopathy[Title/Abstract])) OR (Myocardial Disease[Title/Abstract])) OR (Cerebrovascular Accident[Title/Abstract])) OR (Stokes[Title/Abstract])) OR (CVA[Title/Abstract])) OR (Cerebrovascular Apoplexy[Title/Abstract])) OR (Brain Vascular Accident[Title/Abstract])) OR (Cerebrovascular Stroke[Title/Abstract])) OR (Apoplexy[Title/Abstract])) OR ((((((((((((("Cardiovascular Diseases"[Mesh]) OR "Acute Coronary Syndrome"[Mesh]) OR "Heart Failure"[Mesh]) OR "Coronary Disease"[Mesh]) OR "Atherosclerosis"[Mesh]) OR "Arrhythmias, Cardiac"[Mesh]) OR "Atrial Fibrillation"[Mesh]) OR "Ventricular Fibrillation"[Mesh]) OR "Angina Pectoris"[Mesh]) OR "Myocardial Infarction"[Mesh]) OR "Hypertension"[Mesh]) OR ( "Cardiomyopathies"[Mesh] OR "Diabetic Cardiomyopathies"[Mesh] )) OR "Stroke"[Mesh])) AND ((((((((((Diabetes Mellitus, Noninsulin-Dependent[Title/Abstract]) OR (Non-Insulin-Dependent Diabetes Mellitus[Title/Abstract])) OR (Diabetes Mellitus, Type II[Title/Abstract])) OR (NIDDM[Title/Abstract])) OR (T2DM[Title/Abstract])) OR (Type 2 Diabetes Mellitus[Title/Abstract])) OR (Noninsulin-Dependent Diabetes Mellitus[Title/Abstract])) OR (Type 2 Diabetes[Title/Abstract])) OR (Diabetes, Type 2[Title/Abstract])) OR ("Diabetes Mellitus, Type 2"[Mesh]))) AND (((((((((((((((((((((((((((((((((((((((((((((((((((DPP 4 Inhibitor[Title/Abstract]) OR (DPP 4 Inhibitor[Title/Abstract])) OR (Gliptin[Title/Abstract])) OR (Dipeptidyl Peptidase 4 Inhibitor[Title/Abstract])) OR (Sitagliptin[Title/Abstract])) ) OR (Januvia[Title/Abstract])) OR (Linagliptin[Title/Abstract])) OR (Tradjenta[Title/Abstract])) OR (Vildagliptin[Title/Abstract])) OR (saxagliptin[Title/Abstract])) OR (alogliptin[Title/Abstract])) OR (LC15-0444[Title/Abstract])) OR (Gemigliptin[Title/Abstract])) OR (dutogliptin[Title/Abstract])) OR (anagliptin[Title/Abstract])) OR (evogliptin[Title/Abstract])) OR (gosogliptin[Title/Abstract])) OR (Omarigliptin[Title/Abstract])) OR (teneligliptin[Title/Abstract])) OR (trelagliptin[Title/Abstract])) OR (Glucagon-like peptide-1 receptor agonist[Title/Abstract])) OR (GLP-1 receptor agonist[Title/Abstract])) OR (GLP-1 RAs[Title/Abstract])) OR (incretin mimetic[Title/Abstract])) OR (semaglutide[Title/Abstract])) OR (Liraglutide[Title/Abstract])) OR (rGLP-1 protein[Title/Abstract])) OR (albiglutide[Title/Abstract])) OR (lixisenatide[Title/Abstract])) OR (Exenatide[Title/Abstract])) OR (Exendin 4[Title/Abstract])) OR (dulaglutide[Title/Abstract])) OR (taspoglutide[Title/Abstract])) OR (Sodium Glucose Transporter 2 Inhibitor[Title/Abstract])) OR (SGLT2 Inhibitor[Title/Abstract])) OR (Inhibitor, SGLT2[Title/Abstract])) OR (Gliflozin[Title/Abstract])) OR (SGLT 2 Inhibitor[Title/Abstract])) OR (Canagliflozin[Title/Abstract])) OR (dapagliflozin[Title/Abstract])) OR (empagliflozin[Title/Abstract])) OR (ertugliflozin[Title/Abstract])) OR (ipragliflozin[Title/Abstract])) OR (sotagliflozin[Title/Abstract])) OR (LX4211[Title/Abstract])) OR (tofogliflozin[Title/Abstract])) OR (luseogliflozin[Title/Abstract])) OR (remogliflozin[Title/Abstract])) OR (sergliflozin[Title/Abstract])) OR (("Dipeptidyl-Peptidase IV Inhibitors"[Mesh]) OR "Sodium-Glucose Transporter 2 Inhibitors"[Mesh])) | 4277 |
| #7 | ((((randomly) OR (randomized)) OR (randomization)) OR (random)) OR (RCT) Sort by: Most Recent | 1724422 |
| #8 = #6 AND #7 |  | 1509 |

**Embase**

| #1 | 'dipeptidyl peptidase iv inhibitor':ab,ti OR 'dpp 4 inhibitor':ab,ti OR 'dpp iv inhibitor':ab,ti OR gliptin:ab,ti OR 'dipeptidyl peptidase 4 inhibitor':ab,ti OR 'sitagliptin phosphate':ab,ti OR sitagliptin:ab,ti OR linagliptin:ab,ti OR vildagliptin:ab,ti OR saxagliptin:ab,ti OR alogliptin:ab,ti OR gemigliptin:ab,ti OR 'lc15 0444':ab,ti OR dutogliptin:ab,ti OR anagliptin:ab,ti OR evogliptin:ab,ti OR gosogliptin:ab,ti OR omarigliptin:ab,ti OR teneligliptin:ab,ti OR trelagliptin:ab,ti OR 'glucagon-like peptide-1 receptor agonist':ab,ti OR 'glp-1 receptor agonist':ab,ti OR 'glp-1 ras':ab,ti OR 'incretin mimetic':ab,ti OR semaglutide:ab,ti OR liraglutide:ab,ti OR 'rglp-1 protein':ab,ti OR albiglutide:ab,ti OR lixisenatide:ab,ti OR exenatide:ab,ti OR dulaglutide:ab,ti OR taspoglutide:ab,ti OR 'sodium-glucose transporter 2 inhibitors':ab,ti OR 'sglt2 inhibitor':ab,ti OR 'inhibitor, sglt2':ab,ti OR gliflozin:ab,ti OR 'sglt 2 inhibitor':ab,ti OR canagliflozin:ab,ti OR dapagliflozin:ab,ti OR empagliflozin:ab,ti OR ertugliflozin:ab,ti OR ipragliflozin:ab,ti OR sotagliflozin:ab,ti OR tofogliflozin:ab,ti OR luseogliflozin:ab,ti OR remogliflozin:ab,ti OR sergliflozin:ab,ti | 37840 |
| --- | --- | --- |
| #2 | 'diabetes mellitus, type 2':ab,ti OR 'diabetes mellitus, noninsulin-dependent':ab,ti OR 'non-insulin-dependent diabetes mellitus':ab,ti OR 'diabetes mellitus, type ii':ab,ti OR niddm:ab,ti OR t2dm:ab,ti OR 'type 2 diabetes mellitus':ab,ti OR 'type 2 diabetes':ab,ti OR diabetes:ab,ti | 1048781 |
| **#3** | #1 AND #2 | 27161 |
| #4 | 'cardiovascular diseases':ab,ti OR cvd:ab,ti OR ascvd:ab,ti OR 'major adverse cardiac events':ab,ti OR 'cardiac event':ab,ti OR 'adverse cardiac event':ab,ti OR 'acute coronary syndrome':ab,ti OR 'coronary syndrome, acute':ab,ti OR 'syndrome, acute coronary':ab,ti OR 'heart failure':ab,ti OR 'cardiac failure':ab,ti OR 'heart decompensation':ab,ti OR 'myocardial failure':ab,ti OR hf:ab,ti OR chf:ab,ti OR hfpef:ab,ti OR hfref:ab,ti OR 'coronary disease':ab,ti OR 'coronary heart disease':ab,ti OR atherosclerosis:ab,ti OR atheroscleroses:ab,ti OR atherogenesis:ab,ti OR 'arrhythmias, cardiac':ab,ti OR 'cardiac dysrhythmia':ab,ti OR arrhythmia:ab,ti OR 'atrial fibrillation':ab,ti OR 'ventricular fibrillation':ab,ti OR 'angina pectoris':ab,ti OR stenocardia:ab,ti OR angor:ab,ti OR 'myocardial infarction':ab,ti OR 'infarction, myocardial':ab,ti OR 'cardiovascular stroke':ab,ti OR 'myocardial infarct':ab,ti OR 'heart attack':ab,ti OR hypertension:ab,ti OR 'high blood pressure':ab,ti OR cardiomyopathies:ab,ti OR cardiomyopathy:ab,ti OR stroke:ab,ti OR 'cerebrovascular apoplexy':ab,ti OR 'brain vascular accident':ab,ti OR 'cerebrovascular stroke':ab,ti OR apoplexy:ab,ti | 2344074 |
| **#5** | #3 AND #4 | 5718 |
| **#6** | **randomly** OR **randomized** OR **randomization** OR **random** OR **rct** | **2235047** |
| **#7** | #5 AND #6 | **2122** |

**Web of science**

| #1 | (((((((((((((((((((TS=(Dipeptidyl-Peptidase IV Inhibitors)) OR AB=(DPP 4 Inhibitor)) OR AB=(DPP IV Inhibitor)) OR AB=(Gliptin)) OR AB=(Dipeptidyl Peptidase 4 Inhibitor)) OR AB=(Sitagliptin Phosphate)) OR AB=(Sitagliptin)) OR AB=(Linagliptin)) OR AB=(Vildagliptin)) OR AB=(saxagliptin)) OR AB=(alogliptin)) OR AB=(LC15-0444)) OR AB=(Gemigliptin)) OR AB=(dutogliptin)) OR AB=(anagliptin )) OR AB=(evogliptin)) OR AB=(gosogliptin)) OR AB=(Omarigliptin)) OR AB=(teneligliptin)) OR AB=(trelagliptin) | 15167 |
| --- | --- | --- |
| #2 | ((((((((((((TS=(Glucagon-like peptide-1 receptor agonist)) OR AB=(Glucagon-like peptide-1 receptor agonist)) OR AB=(GLP-1 receptor agonist)) OR AB=(GLP-1 RAs)) OR AB=(incretin mimetic)) OR AB=(semaglutide)) OR AB=(Liraglutide)) OR AB=(rGLP-1 protein)) OR AB=(albiglutide)) OR AB=(lixisenatide)) OR AB=(Exenatide)) OR AB=(dulaglutide)) OR AB=(taspoglutide) | 14992 |
| #3 | (((((((((((((((TS=(Sodium-Glucose Transporter 2 Inhibitors)) OR AB=(Sodium Glucose Transporter 2 Inhibitor)) OR AB=(SGLT2 Inhibitor)) OR AB=(Inhibitor, SGLT2)) OR AB=(Gliflozin)) OR AB=(SGLT 2 Inhibitor)) OR AB=(SGLT 2 Inhibitor)) OR AB=(dapagliflozin)) OR AB=(empagliflozin)) OR AB=(ertugliflozin)) OR AB=(ipragliflozin)) OR AB=(sotagliflozin)) OR AB=(tofogliflozin)) OR AB=(tofogliflozin)) OR AB=(remogliflozin)) OR AB=(sergliflozin) | 12459 |
| #4 | #1 OR #2 OR #3 | 37760 |
| #5 | ((((((((TS=(Diabetes Mellitus, Type 2)) OR AB=(Diabetes Mellitus, Noninsulin-Dependent)) OR AB=(Non-Insulin-Dependent Diabetes Mellitus)) OR AB=(Diabetes Mellitus, Type II)) OR AB=(NIDDM)) OR AB=(T2DM)) OR AB=(Type 2 Diabetes Mellitus)) OR AB=(Type 2 Diabetes)) OR AB=(Diabetes) | 822368 |
| #6 | ((((((((((((((((((((((((((((((((((((((((((((((((((((((TS=(Cardiovascular Diseases)) OR TS=(Acute Coronary Syndrome)) OR TS=(Heart Failure)) OR TS=(Coronary Disease)) OR TS=(Atherosclerosis)) OR TS=(Arrhythmias, Cardiac)) OR TS=(Atrial Fibrillation)) OR TS=(Ventricular Fibrillation)) OR TS=(Angina Pectoris)) OR TS=(Myocardial Infarction)) OR TS=(Hypertension)) OR TS=(Cardiomyopathies)) OR TS=(Stroke)) OR AB=(Cardiovascular disease)) OR AB=(CVD)) OR AB=(ASCVD)) OR AB=(Major Adverse Cardiac Events)) OR AB=(Cardiac Event)) OR AB=(Adverse Cardiac Event)) OR AB=(Coronary Syndrome, Acute)) OR AB=(Syndrome, Acute Coronary))) OR AB=(Cardiac Failure)) OR AB=(Heart Decompensation)) OR AB=(Myocardial Failure)) OR AB=(HF)) OR AB=(CHF)) OR AB=(HFpEF)) OR AB=(HFrEF)) OR AB=(Coronary Heart Disease)) OR AB=(Atheroscleroses)) OR AB=(Atherogenesis)) OR AB=(Cardiac Dysrhythmia)) OR AB=(Arrhythmia)) OR AB=(Atrial Fibrillations)) OR AB=(Fibrillation)) OR AB=(Ventricular Fibrillations)) OR AB=(Stenocardia)) OR AB=(Angor)) OR AB=(Infarction, Myocardial)) OR AB=(Cardiovascular Stroke)) OR AB=(Myocardial Infarct)) OR AB=(Heart Attack)) OR AB=(High Blood Pressure)) OR AB=(Cardiomyopathy)) OR AB=(Myocardiopathy)) OR AB=(Myocardial Disease)) OR AB=(Stokes)) OR AB=(Cerebrovascular Accident)) OR AB=(CVA)) OR AB=(Cerebrovascular Apoplexy)) OR AB=(Brain Vascular Accident)) OR AB=(Cerebrovascular Stroke)) OR AB=(Apoplexy)) | 4410266 |
| #7 | ((((AB=(randomly )) OR AB=(randomized )) OR AB=(randomization )) OR AB=(random)) OR AB=(RCT) | 2520372 |
| #8 | #4 AND #5 AND #6 AND #7 | 2453 |

**Cochrane library**

| #1 | MeSH descriptor: [Dipeptidyl-Peptidase IV Inhibitors] explode all trees | 747 |
| --- | --- | --- |
| #2 | (DPP 4 Inhibitor):ti,ab,kw OR (DPP IV Inhibitor):ti,ab,kw OR (Gliptin):ti,ab,kw OR (Dipeptidyl Peptidase 4 Inhibitor):ti,ab,kw | 1637 |
| #3 | (Sitagliptin Phosphate):ti,ab,kw OR (Sitagliptin):ti,ab,kw OR (Linagliptin):ti,ab,kw OR (Vildagliptin):ti,ab,kw OR (saxagliptin):ti,ab,kw | 4115 |
| #4 | (alogliptin):ti,ab,kw OR (LC150444):ti,ab,kw OR (dutogliptin):ti,ab,kw OR (anagliptin):ti,ab,kw OR (evogliptin):ti,ab,kw | 444 |
| #5 | (evogliptin):ti,ab,kw OR (evogliptin):ti,ab,kw OR (Omarigliptin):ti,ab,kw OR (teneligliptin):ti,ab,kw OR (trelagliptin):ti,ab,kw | 316 |
| #6 | #1 OR #2 OR #3 OR #4 OR #5 | 5907 |
| #7 | (Glucagon-like peptide-1 receptor agonist):ti,ab,kw OR (GLP-1 receptor agonist):ti,ab,kw OR (GLP-1 RAs):ti,ab,kw OR (incretin | 1601 |
| #8 | (semaglutide):ti,ab,kw OR (Liraglutide):ti,ab,kw OR (rGLP 1 protein):ti,ab,kw OR (albiglutide):ti,ab,kw OR (lixisenatide):ti,ab,kw | 3767 |
| #9 | (Exenatide):ti,ab,kw OR (Exendin 4):ti,ab,kw OR (dulaglutide):ti,ab,kw OR (taspoglutide):ti,ab,kw | 1992 |
| #10 | #7 OR #8 OR #9 | 5804 |
| #11 | MeSH descriptor: [Sodium-Glucose Transporter 2 Inhibitors] explode all trees | 774 |
| #12 | (Sodium Glucose Transporter 2 Inhibitor):ti,ab,kw OR (SGLT2 Inhibitor):ti,ab,kw OR (Inhibitor, SGLT2):ti,ab,kw OR (Gliflozin):ti,ab,kw  OR (SGLT 2 Inhibitors):ti,ab,kw (Canagliflozin):ti,ab,kw OR (dap | 1846 |
| #13 | (Canagliflozin):ti,ab,kw OR (dapagliflozin):ti,ab,kw OR (empagliflozin):ti,ab,kw OR (ertugliflozin):ti,ab,kw OR (ipragliflozin):ti,ab,kw | 4828 |
| #14 | (sotagliflozin):ti,ab,kw OR (tofogliflozin):ti,ab,kw OR (luseogliflozin):ti,ab,kw OR (remogliflozin):ti,ab,kw OR (sergliflozin):ti,ab,kw | 378 |
| #15 | #11 OR #12 OR #13 OR #14 | 5532 |
| #16 | #6 OR #10 OR #15 | 14783 |
| #17 | MeSH descriptor: [Diabetes Mellitus, Type 2] explode all trees | 23568 |
| #18 | (Diabetes Mellitus, Noninsulin Dependent):ti,ab,kw OR (Diabetes Mellitus, Type II):ti,ab,kw OR (NIDDM):ti,ab,kw OR (T2DM):ti,ab,kw  OR (Type 2 Diabetes Mellitus):ti,ab,kw |  |
| #19 | (Type 2 Diabetes):ti,ab,kw OR (Diabetes):ti,ab,kw |  |
| #20 | #17 0R #18 OR #19 | 104210 |
| #21 | MeSH descriptor: [Cardiovascular Diseases] explode all trees | 152906 |
| #22 | (Cardiovascular Diseases):ti,ab,kw OR (CVD):ti,ab,kw OR (ASCVD):ti,ab,kw OR (Major Adverse Cardiac Events):ti,ab,kw OR (Cardiac  Event):ti,ab,kw | 39118 |
| #23 | MeSH descriptor: [Acute Coronary Syndrome] explode all trees | 3322 |
| #24 | (Coronary Syndrome, Acute):ti,ab,kw OR (Syndrome, Acute Coronary):ti,ab,kw | 8157 |
| #25 | MeSH descriptor: [Heart Failure] explode all trees | 14723 |
| #26 | (Cardiac Failure):ti,ab,kw OR (Heart Decompensation):ti,ab,kw OR (Myocardial Failure):ti,ab,kw OR (HF):ti,ab,kw OR (CHF):ti,ab,kw | 34582 |
| #27 | (HFpEF):ti,ab,kw OR (HFrEF):ti,ab,kw | 2216 |
| #28 | MeSH descriptor: [Coronary Disease] explode all trees | 18548 |
| #29 | MeSH descriptor: [Atherosclerosis] explode all trees | 4498 |
| #30 | MeSH descriptor: [Arrhythmias, Cardiac] explode all trees | 14339 |
| #31 | (Coronary Heart Disease):ti,ab,kw OR (Atheroscleroses):ti,ab,kw OR (Atherogenesis):ti,ab,kw OR (Cardiac Dysrhythmia):ti,ab,kw OR  (Arrhythmia):ti,ab,kw | 34314 |
| #32 | MeSH descriptor: [Atrial Fibrillation] explode all trees | 7504 |
| #33 | MeSH descriptor: [Ventricular Fibrillation] explode all trees | 630 |
| #34 | MeSH descriptor: [Angina Pectoris] explode all trees | 5589 |
| #35 | (Atrial Fibrillations):ti,ab,kw OR (Fibrillation):ti,ab,kw OR (Ventricular Fibrillations):ti,ab,kw OR (Stenocardia):ti,ab,kw | 18035 |
| #36 | MeSH descriptor: [Myocardial Infarction] explode all trees | 14055 |
| #37 | (Infarction, Myocardial):ti,ab,kw OR (Cardiovascular Stroke):ti,ab,kw OR (Myocardial Infarct):ti,ab,kw OR (Heart Attack):ti,ab,kw | 44073 |
| #38 | MeSH descriptor: [Hypertension] explode all trees | 26767 |
| #39 | MeSH descriptor: [Cardiomyopathies] explode all trees | 2505 |
| #40 | (High Blood Pressure):ti,ab,kw OR (Cardiomyopathy):ti,ab,kw OR (Myocardiopathy):ti,ab,kw OR (Myocardial Disease):ti,ab,kw | 55818 |
| #41 | MeSH descriptor: [Stroke] explode all trees | 15322 |
| #42 | (Cerebrovascular Accident):ti,ab,kw OR (CVA):ti,ab,kw OR (Cerebrovascular Apoplexy):ti,ab,kw OR (Brain Vascular Accident):ti,ab,kw  OR (Cerebrovascular Stroke):ti,ab,kw | 21243 |
| #43 | (Stokes):ti,ab,kw OR (Apoplexy):ti,ab,kw | 625 |
| #44 | #21 OR #22 OR #23 OR #24 OR #25 OR #26 OR #27 OR #28 OR #29 OR #30 OR #31 OR #32 OR #33 OR #34 OR #35 OR #36 OR #37 OR #38 OR #39 OR #40 OR #41 OR #42 OR #43 | 241793 |
| #45 | (randomly):ti,ab,kw OR (randomized):ti,ab,kw OR (randomization):ti,ab,kw OR (random):ti,ab,kw OR (RCT):ti,ab,kw | 1287623 |
| #46 | (#16) AND (#20) AND (#44) AND (#45) | 2056 |


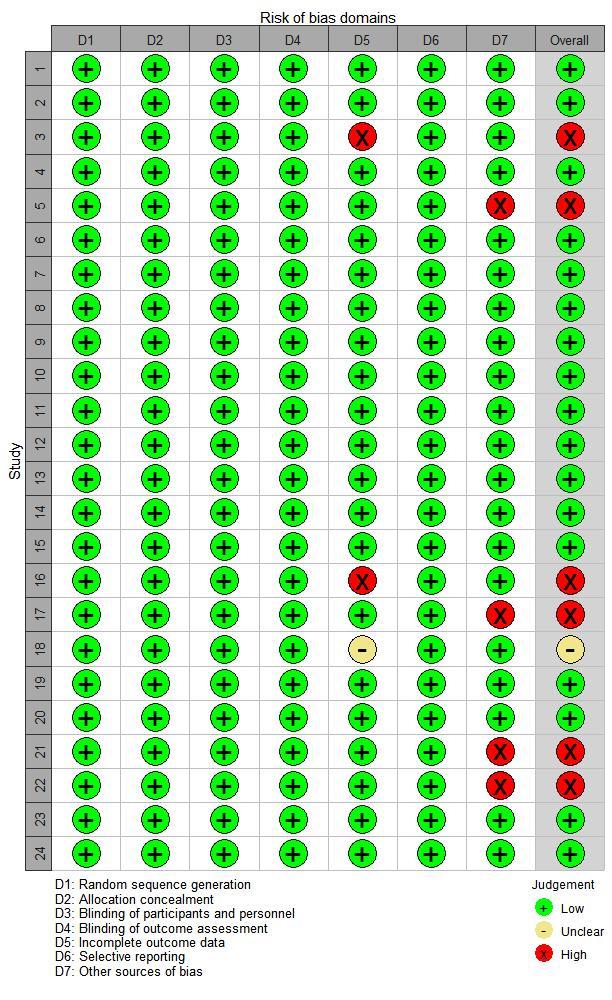


Supplementary Figure 1 Total plot of risk of bias for literature quality evaluation

(1：NCT01107886，2：NCT00790205，3：NCT00968708，4：NCT00894868，5：NCT01703208，6：NCT01897532，7：NCT02465515，8：NCT01179048，9：NCT03496298，10：NCT01394952，11：NCT02692716，12：NCT01147250，13：NCT01455896，14：NCT01720446，15：NCT01144338，16：NCT01986881，17：NCT01032629和NCT01989754，18：NCT01730534，19：NCT03036124，20：NCT01131676，21：NCT03521934，22：NCT03315143，23：NCT03057977，24:NCT02065791.)


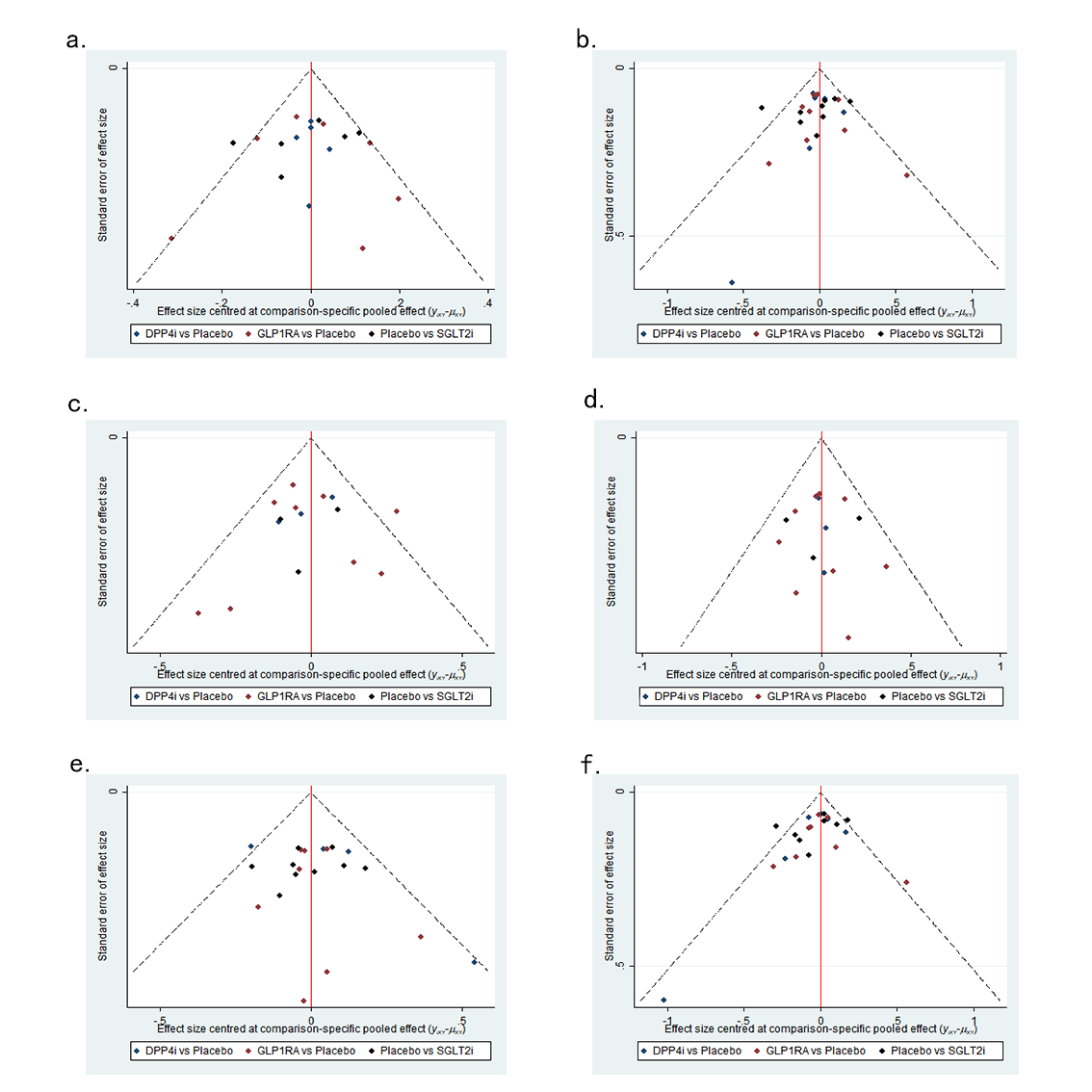


Supplementary Figure 2. Comparison-adjusted funnel plot. a.Comparison-adjusted funnel plot of MACE. b.Comparison-adjusted funnel plot of Cardiovascular death. c.Comparison-adjusted funnel plot of nonfatal myocardial infarction. d.Comparison-adjusted funnel plot of nonfatal stoke. e.Comparison-adjusted funnel plot of HHF. f.Comparison-adjusted funnel plot of all-cause mortality.
